# Mathematical Framework to Identify Optimal Molecule Based on Virtual Ligand Strategy

**DOI:** 10.1021/acs.jcim.5c00815

**Published:** 2025-06-13

**Authors:** Wataru Matsuoka, Ken Hirose, Ren Yamada, Taihei Oki, Satoru Iwata, Satoshi Maeda

**Affiliations:** † Institute for Chemical Reaction Design and Discovery (WPI-ICReDD), 12810 Hokkaido University, Kita 21, Nishi 10, Kita-ku, Sapporo, Hokkaido 001-0021, Japan; ‡ JST, ERATO Maeda Artificial Intelligence in Chemical Reaction Design and Discovery Project, Kita 10, Nishi 8, Kita-ku, Sapporo, Hokkaido 060-0810, Japan; § Department of Chemistry, Faculty of Science, Hokkaido University, Kita 10, Nishi 8, Kita-ku, Sapporo, Hokkaido 060-0810, Japan; ∥ Graduate School of Chemical Sciences and Engineering, Hokkaido University, Kita 13, Nishi 8, Kita-ku, Sapporo, Hokkaido 060-8628, Japan; ⊥ Department of Mathematical Informatics, Graduate School of Information Science and Technology, 13143 The University of Tokyo, Hongo 7-3-1, Bunkyo-ku, Tokyo 113-8656, Japan

## Abstract

Identifying molecular entities with desired properties from a vast pool of potential candidates is a fundamental challenge in organic chemistry. In particular, ligand engineering—designing optimal ligands for transition metal catalysis—has been extensively studied over the past few decades. To address this challenge, we previously proposed the virtual ligand (VL) approach, a computational method that introduces a mathematical model to approximate ligand molecules within quantum chemical calculations. This model is then optimized to identify the electronic and steric properties most suited for a given reaction. However, the interpretability of the resulting VL parameters remained elusive, limiting predictions to a qualitative level. In this study, we establish a mathematical framework that links real molecules to the VL parameters, thereby enabling rapid and quantitative prediction of optimal ligands. The prediction algorithm was validated across four different reactions, and its accuracy, limitations and potential improvements are discussed.

## Graphical abstract

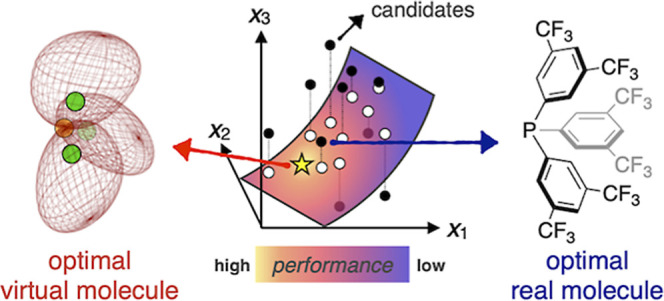

## 1 Introduction

Identifying molecular entities that exhibit desired properties, such as high catalytic activity, photophysical characteristics, and biological activity, from a vast array of candidate molecules is a central challenge in organic chemistry. For instance, identifying an optimal ligand that exhibits appropriate electronic and steric effects for a desired chemical transformation—or ligand engineering—is particularly important in the development of transition metal catalysis.^1–7^ Traditionally, this process has relied heavily on experimental trial-and-error, frequently resulting in significant waste of time and resources. To streamline this process, various in silico approaches, including high-throughput computations and data-driven candidate selection, have been explored.^8–19^ Recently, we introduced a novel computational approach based on the virtual ligand (VL).^20–26^ A virtual ligand (VL) is a mathematically modeled entity designed to approximate the electronic and steric properties of real ligands in quantum chemical calculations. Here, the “real” ligand refers to the full molecular system, analogous to the real system in QM/MM calculations. While the electronic and steric properties of real ligands are intrinsically determined by their chemical structure, those of a VL can be independently adjusted by modifying parameters in its electronic and steric approximation. Leveraging this flexibility, two strategies—namely virtual ligand-assisted screening (VLAS)^20^ and virtual ligand-assisted optimization (VLAO)^23^—were developed to identify the electronic and steric properties of the optimal ligand (Figure 1a). In the VLAS approach, the entire parameter space is systematically explored using a grid search method to identify the optimal parameter set that maximizes (or minimizes) the given objective function. In contrast, the VLAO approach analytically differentiates the objective function with respect to the electronic and steric parameters. The optimal parameter set is then identified through gradient-driven numerical optimization, a scheme analogous to geometry optimization using analytical energy gradients. Regardless of the approach, the obtained optimal parameter set is expected to represent the electronic and steric properties of the optimal ligand for a given reaction, providing a rational guideline for ligand design. Indeed, using this strategy, we have identified high-performing ligands both theoretically and experimentally for various reactions, including hydroformylation, Suzuki–Miyaura cross-coupling, and hydrogermylation.^20–23^ However, because the VL parameters were only qualitatively interpretable (e.g., “electron-rich” or “bulky” compared to PPh_3_), designing real ligand molecules still relied on prior knowledge and experience.

**Figure 1:**
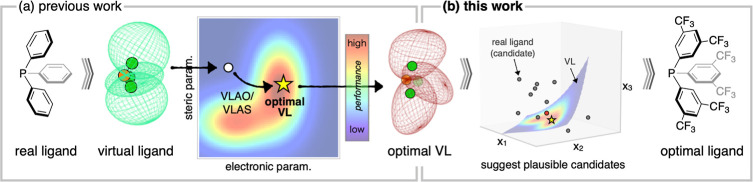
Schematic illustration of the VL strategy. (a) Optimization of VL parameters using the VLAS and VLAO approaches (previous work). (b) A method for selecting real molecules based on optimal VL parameters (this work).

Here, we introduce a mathematical framework for selecting real molecules based on the VL parameters (Figure 1b). To achieve this, we first provide a mathematical interpretation of the relationship between VLs and real ligands, which was previously ambiguous and qualitative. This, in turn, enables the quantitative prediction of the performance of real ligands by comparing the parameters of the optimal VL with those corresponding to real ligands. The notable feature of this method is that the relationship between VLs and real ligands is independent of the target reaction, enabling the creation of a database for VL parameters corresponding to real ligands. This allows for quantitative performance predictions of real ligands using simple quantum chemical calculations with VLs, without the need for experimental or computational studies involving real ligands. The prediction algorithm has been validated in four reactions, including two internal validations and two external validations, where monodentate phosphine ligands are optimized to minimize or maximize reaction/activation energies. Through these demonstrations, we discuss the reliability, limitations, and potential improvements.

## 2 Theory and Method

### 2.1 Mathematical Model

We model the system as follows: let *R* and *P* represent the sets of all real candidate molecules and all possible virtual ligands, respectively. While *R* is a finite set (i.e., candidate molecules), *P* is a subset of $R^{n}$ , where each element is a VL parameter $p\in R^{n}$ . For any ligand, whether virtual or real, a scalar value *y* that represents the performance of a ligand can be computed based on energies of equilibrium structures (EQs) and transition states (TSs). Examples of *y* include the activation energy, reaction rate and selectivity estimated within the framework of the transition state theory (TST), as well as the computed product yield derived from the reaction path network.^27–30^ Similarly, an *m*-dimensional descriptor vector $x\in R^{m}$ (*m* > *n*) can also be calculated for both virtual and real molecules (see subsection [Sec sec2.2] for **x** used in this study). Computations of *y* and **x** for virtual and real molecules are denoted by the following functions

$$F_{real}:R\to Y$$

$$F_{virt}:P\to Y$$

$$G_{real}:R\to X$$

$$G_{virt}:P\to X$$

where Y⊆R and $X\subseteq R^{m}$ are the sets of all possible *y* and **x**, respectively. Figure 2 summarizes these notions. Note that *F*_real_ is equivalent to the conventional computational study using real ligands, where the performance of each ligand candidate is estimated through investigation of the potential energy surface (PES).^31–35^

**Figure 2:**
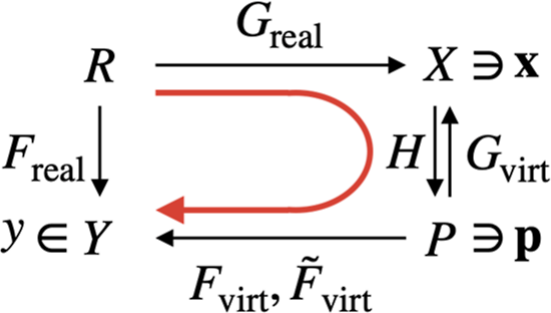
Mathematical model discussed in this study. The functions *H* and ${\mathrm{F̃}}_{virt}$ are defined in Subsections [Sec sec2.3] and 2.4, respectively.

The discussion below is based on the following assumptions: (1) functions *F*_virt_ and *G*_virt_ are smooth with respect to the VL parameter **p**, and (2) each component of **x** is uncorrelated with the others. The first criterion can be satisfied by defining *F*_virt_ and *G*_virt_ as differentiable functions of energies of EQs and TSs computed using VLs, because these energies are differentiable in terms of **p**, as previously reported.^23^ The second requirement for **x** can be easily satisfied by taking a linear combination of such computed energies to eliminate the correlation (see Subsection [Sec sec2.2] for details).

The problem of identifying the optimal ligand for the reaction can be formulated as follows

$$Maximize\;F_{real}\left(i\right)\;subject\;to\;i\in R$$

If *F*_real_(*R*) (i.e., the set of values of *F*_real_ for all the elements in *R*) can be obtained by the brute-force search, the optimal ligand for the reaction can be easily identified by selecting a molecule *i* ∈ *R* that maximizes *y* = *F*_real_(*i*). However, due to the high computational cost of quantum chemical calculations and the vast diversity of real ligand molecules, obtaining *F*_real_(*R*) by individually exploring the PES for all candidates is impractical. Therefore, the purpose of this study is to find elements of *R* which are most likely to exhibit the best *y* value without evaluating *F*_real_. To achieve this in a mathematically reasonable and computationally efficient manner, we introduce the following two functions

H:X→P

$${\mathrm{F̃}}_{virt}:P\to Y$$

Here, *H* is a mapping that projects the descriptor vector **x** onto the set of VL parameters *P*, and ${\mathrm{F̃}}_{virt}$ is the second-order Taylor expansion of *F*_virt_. More details of *H* and ${\mathrm{F̃}}_{virt}$ are described in Subsections [Sec sec2.3] and 2.4 later, respectively. With these functions, *F*_real_(*i*) can be approximated by calculating ${\mathrm{F̃}}_{virt}\left(H\left(G_{real}\left(i\right)\right)\right)$ (see the red arrow in Figure 2).

### 2.2 Descriptor Vector **x** and the Functions *G*_real_ and *G*_virt_

The descriptor vector **x** should encapsulate the electronic and steric characteristics of ligand molecules while satisfying the two criteria described in the previous subsection (differentiability and uncorrelation of components). In this study, considering the successful descriptors in the ligand knowledge base (LKB),^36–40^ ligand dissociation energies of *m* arbitrary complexes (i.e., reaction energies for L + Fg_*i*_ → LFg_*i*_, where L is the ligand and Fg_*i*_ represents the remaining fragment of *i*-th complex) were used to construct an *m*-dimensional descriptor vector **x**. However, other types of physical quantities, including activation energies for representative elementary steps, may also be used for descriptor construction, as long as they capture relevant ligand properties and are differentiable with respect to the VL parameters. To ensure the uncorrelation of the descriptor components, principal component analysis (PCA) was performed on the dissociation energies for all real ligand candidates, and **x** was determined as the *m* principal component scores. Thus, the function *G*_real_ is equivalent to performing quantum chemical calculations for the *m* complexes with a given real ligand, followed by a linear transformation of the dissociation energies based on the PCA transformation matrix. Similarly, the function *G*_virt_ is equivalent to performing quantum chemical calculations for the *m* complexes using a VL with a given parameter **p**, followed by a linear transformation of the calculated dissociation energies. As the dissociation energies calculated with the VL can be differentiated with respect to the VL parameter **p**,^23^ the derivative of the function *G*_virt_ can also be computed.

### 2.3 Definition of *H*

The function *H* is a transformation that returns the parameter **p** from a given descriptor vector **x**. Here, **x** lies in the *m*-dimensional space. On the other hand, since *P* is a subset of the *n*-dimensional space, *G*_virt_(*P*) forms an *n*-dimensional surface embedded in the *m*-dimensional space (Figure 3). Thus, *H* is a map from the *m*-dimensional space onto the *n*-dimensional surface. In this study, we defined *H* as a function that projects **x** to the nearest point on *G*_virt_(*P*), returning the corresponding VL parameter **p**. The function can be formulated as follows

$$H\left(x\right)≔\arg\min_{p\in P}l\left(x,p\right)$$

where *l*(**x**,**p**) denotes the Euclidean distance between **x** and *G*_virt_(**p**). Since the position in the *m*-dimensional space represents the electronic and steric properties of the ligand, the smaller the *l*(**x**,**p**), the more similar the electronic and steric properties of the corresponding ligands. Therefore, *H*(*G*_real_(*i*)) represents the parameter **p** for the VL that best represents the given real ligand *i* ∈ *R*. In practice, *H*(**x**) can be determined by the VLAO method, where **p** is numerically optimized to minimize *l*(**x**,**p**).

**Figure 3:**
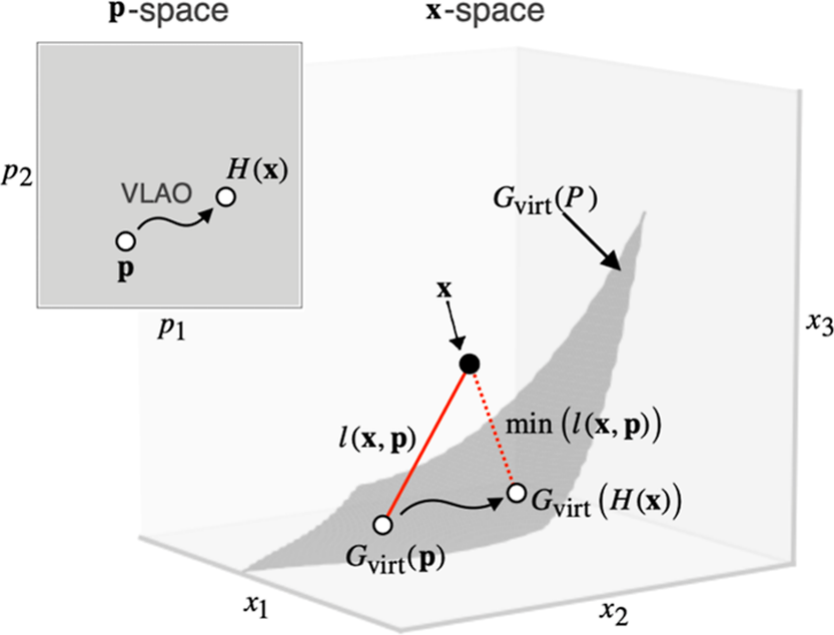
Schematic illustration of the relationship between the *n*-dimensional parameter space (**p**-space) and *m*-dimensional descriptor space (**x**-space). For clarity, *n* and *m* are set to 2 and 3, respectively.

### Definition of ${\mathrm{F̃}}_{virt}$

2.4

The function *F*_virt_ evaluates the performance *y* of the VL with given parameter **p** based on quantum chemical calculations. Generally, the function *F*_virt_ is much less computationally demanding than *F*_real_, because quantum chemical calculations using VLs are much faster than those using corresponding real ligands due to the reduced number of electrons and conformational flexibility. However, when the size of *R* (the number of candidate molecules) increases significantly, obtaining *F*_virt_ for each candidate molecule via numerous quantum chemical calculations can become a bottleneck in the overall process (see Subsection [Sec sec2.5] for details). Therefore, it is desirable to use a model function ${\mathrm{F̃}}_{virt}$ that approximates *F*_virt_. In this study, ${\mathrm{F̃}}_{virt}$ was defined as the second-order Taylor expansion of *F*_virt_, as follows

$${\mathrm{F̃}}_{virt}\left(p\right)=F_{virt}\left(p^{*}\right)+\left(\frac{dF_{virt}}{dp}\left(p^{*}\right)\right)\Delta p+\frac{1}{2}\Delta p^{T}\left(\frac{d^{2}F_{virt}}{dp^{2}}\left(p^{*}\right)\right)\Delta p$$

where p* is a reference point of the Taylor expansion, and Δ**p** is the difference vector between **p** and p* (Figure 4). For the derivation of the second-order derivative, see Supporting Information. Since the main purpose of this study is to identify several plausible candidates, rather than to accurately predict *y* values for all candidates, the reference point for the Taylor expansion should be as close as possible to the maximum (or minimum) point of the function *F*_virt_. As previously demonstrated, such parameters can be readily obtained using the VLAO method for various objectives, including the activation barrier, regioselectivity and enantioselectivity of the target reaction.^23^ Once p* is determined, the function ${\mathrm{F̃}}_{virt}$ can be readily defined, enabling us to estimate the *y* value for a given **p** without performing any quantum chemical calculations. After examining the reliability of ${\mathrm{F̃}}_{virt}$ and its limitations, another model function to approximate *F*_virt_, as well as the use of *F*_virt_ itself, will be discussed in the later part of this paper (Subsection [Sec sec3.6]).

**Figure 4:**
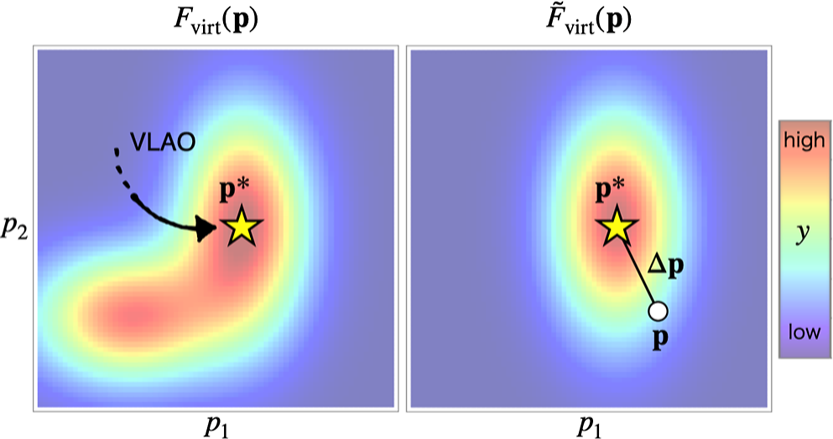
Schematic illustration of the model function ${\mathrm{F̃}}_{virt}$ in an *n*-dimensional parameter space. For clarity, *n* is set to 2.

### 2.5 Overall Process

To summarize the discussion above, the performance of all candidate molecules (i.e., *F*_real_(*R*)) can be approximated by calculating ${\mathrm{F̃}}_{virt}\left(H\left(G_{real}\left(R\right)\right)\right)$ . The function *G*_real_ is an operation to prepare the descriptor vector **x** for a given real ligand, where quantum chemical calculations of ligand dissociation energies followed by a linear transformation of the resulting energies are performed. The function *H* is equivalent to the VLAO calculation to identify the VL parameter **p** which best reproduces a given descriptor **x**. The function ${\mathrm{F̃}}_{virt}$ is the second-order Taylor expansion of *F*_virt_ around its extremum, thus providing a good approximation of *F*_virt_ for a given VL parameter **p**. It should be noted that although obtaining *H*(*G*_real_(*R*)) (i.e., calculating **p** for all candidate molecules) may appear computationally demanding—especially as the number of candidate molecules increases (i.e., the size of *R* increases)—*H*(*G*_real_(*R*)) is independent of the reaction of interest. Therefore, if a parameter database containing the VL parameters **p** for all candidate molecules has been constructed in advance, the computational cost to obtain *H*(*G*_real_(*R*)) becomes negligible. Consequently, once the VLAO calculation to maximize the performance *y* of the VL in the target reaction has been performed, ${\mathrm{F̃}}_{virt}\left(H\left(G_{real}\left(R\right)\right)\right)$ can be computed instantaneously, without any additional quantum chemical calculations.

### 2.6 Virtual Ligand Used in This Study

In this paper, we adopted a previously developed VL^23^ for all calculations. In this virtual ligand, substituents in a phosphorus­(III) ligand PR_3_ are replaced with Cl atoms subjected to three penalty functions: the keep potential, the keep angle potential and the ovoid-based Lennard-Jones (LJ) potential. The resulting PCl*_3_ is used for quantum chemical calculations (Figure 5). The keep potential and the keep angle potential are harmonic potentials applied to each P–Cl distance and Cl–P–Cl angle, respectively. These penalty functions primarily serve to approximate electronic effects. By tuning the equilibrium distance *r*_0_ and the equilibrium angle ϕ_0_ in these functions, the electronic properties of PCl*_3_ can be modified. The ovoid-based LJ potential (hereafter, simply denoted as the ovoid LJ) is a penalty function to approximate steric effect. By defining an ovoid along with a P–Cl* axis and computing the LJ potential based on it, steric interactions can be approximated. The width, thickness and length of the ovoid as well as its distance from the P atom are determined by seven geometric parameters: *a*_1_, *a*_2_, *b*_1_, *b*_2_, *c*_1_, *c*_2_ and *d*. The steric properties of the VL can be modified by tuning these parameters. For a more detailed description of the implementation, see the previous work.^23^ It should be noted that, in this study, considering the ease of convergence in numerical optimization and the high interpretability of the results, only three essential parameters—*r*_0_ for the keep potential and *a*_1_, *b*_1_ for the ovoid LJ potential—were used as variables. ϕ_0_ for the keep angle potential, *c*_1_, *c*_2_ and *d* for the ovoid LJ potential were kept constant at 65°, 3.0 Å, 3.0 Å and 3.0 Å, respectively. *a*_2_ and *b*_2_ for the ovoid LJ potential were set to be the same as *a*_1_ and *b*_1_ so that the ovoid represents a symmetrical substituent.

**Figure 5:**
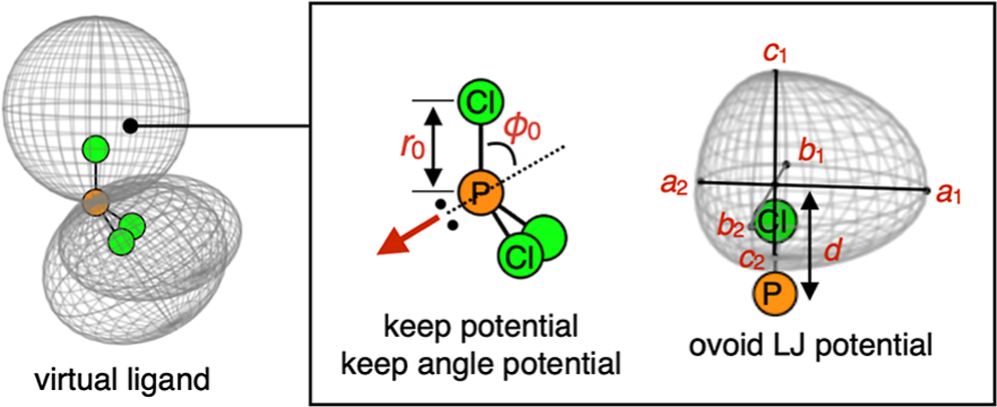
Penalty functions of the VL used in this study and its parameters.

### 2.7 Computational Details

All energy and gradient calculations at the density functional theory (DFT) level were performed using the Gaussian 16 software.^41^ Geometry optimizations were conducted with the GRRM23 software^42^ in conjunction with the VL program, which is available at https://github.com/WatMat1127/V2R. All DFT calculations were performed using the ωB97X-D functional and the Def2-SVP basis set. Detailed computational procedures are provided in the Supporting Information.

## 3 Result and Discussion

### 3.1 Development of Parameter Database

As described in the Subsection [Sec sec2.6], a parameter database, *H*(*G*_real_(*R*)), can be constructed independently of target reactions, thereby accelerating the overall process of calculating ${\mathrm{F̃}}_{virt}\left(H\left(G_{real}\left(R\right)\right)\right)$ . In this study, ligand dissociation energies of 16 diverse complexes, shown in Figure 6, were calculated to prepare descriptor vector **x**. These complexes were arbitrarily selected, considering variation in metal element, oxidation state and steric environment around the metal center, as well as the robustness of the quantum chemical calculations, ensuring that the resulting descriptor vector captures the electronic and steric properties as comprehensively as possible. A set of 21 monodentate phosphine ligands was considered as *R*, and the dissociation energies for all 336 complexes (21 ligands × 16 fragments) were computed. PCA was performed on the resulting dissociation energies, and the descriptor vector **x** for all ligands (i.e., *G*_real_(*R*)) was calculated. The dissociation energies and **x** for each ligand are summarized in Supporting Information. Subsequently, the VLAO calculation to minimize *l*(**x**,**p**) were performed for all ligands, and the corresponding VL parameters (i.e., *H*(*G*_real_(*R*))) were calculated. In the VLAO calculation, rather than minimizing *l*(**x**,**p**) directly, *l*(**x**,**p**)^2^ were minimized. Starting from an initial parameter set **p** = (*r*_0_,*a*_1_,*b*_1_)=(1.6 Å, 3.0 Å, 2.0 Å), optimization was carried out using the conjugate gradient (CG) method. The resulting VL parameters corresponding to each real ligand, along with its residual *l*(**x**,**p**) value (denoted as *l*_min_), are summarized in Table 1. This value indicates how similar a given real ligand and the corresponding VL are in terms of electronic and steric properties. As shown in the following discussions, this quantity can be utilized as a metric to estimate the reliability of ${\mathrm{F̃}}_{virt}\left(H\left(G_{real}\left(R\right)\right)\right)$ as an approximation of *F*_real_(*R*).

**Figure 6:**
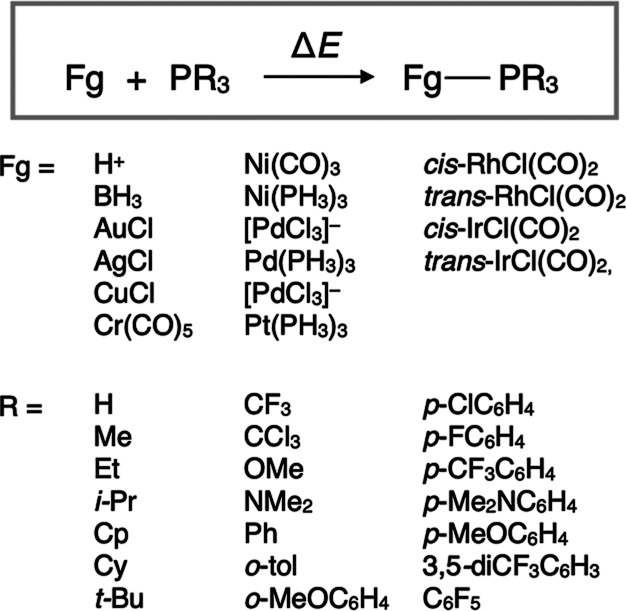
Ligand dissociation energies used to derive descriptor vector **x**.

**Table 1:** VL Parameters Optimized to Reproduce Real Ligands and Corresponding *l*_min_ Values

	ligand	*r*_0_ (Å)	*a*_1_ (Å)	*b*_1_ (Å)	*l*_min_
L1	P(3,5-diCF_3_C_6_H_3_)_3_	1.685	4.307	1.225	3.968
L2	P(C_6_F_5_)_3_	1.857	2.795	1.490	2.261
L3	P(CCl_3_)_3_	1.966	2.995	2.119	3.330
L4	P(CF_3_)_3_	2.032	2.460	1.327	2.699
L5	PCp_3_	1.160	5.007	1.231	2.916
L6	PCy_3_	1.236	4.181	1.415	2.366
L7	PEt_3_	1.424	2.620	1.364	1.419
L8	PH_3_	1.898	3.132	2.140	4.614
L9	PMe_3_	1.557	2.132	0.608	1.112
L10	P(NMe_2_)_3_	1.418	2.880	1.846	1.488
L11	P(OMe)_3_	1.637	2.555	1.418	1.482
L12	PPh_3_	1.569	2.873	1.816	1.744
L13	P*i*-Pr_3_	1.330	2.679	1.420	2.193
L14	P(*o*-MeOC_6_H_4_)_3_	1.366	2.865	1.840	2.948
L15	P(*o*-tol)_3_	1.669	4.653	1.640	3.094
L16	P(*p*-CF_3_C_6_H_4_)_3_	1.656	2.667	1.421	2.437
L17	P(*p*-ClC_6_H_4_)_3_	1.618	2.744	1.553	2.020
L18	P(*p*-FC_6_H_4_)_3_	1.594	2.767	1.592	1.863
L19	P(*p*-Me_2_NC_6_H_4_)_3_	1.398	2.985	2.258	2.045
L20	P(*p*-MeOC_6_H_4_)_3_	1.494	2.892	1.874	1.840
L21	P*t*-Bu_3_	1.415	4.863	1.974	2.282

### 3.2 Internal Validation

With the parameter database for 21 phosphine ligands in hand, we conducted an internal validation of the prediction algorithm. Two complexes were selected from the 16 complexes shown in Figure 6, and the objective value (*y*) was defined as the difference between their ligand dissociation energies. This objective value corresponds to the reaction energy for the formal transmetalation of a given ligand from one metal fragment to the other (i.e., ${LFg}_{i}+{Fg}_{j}\to {{Fg}_{i}+LFg}_{j}$ ). Although such reactions may not have significant chemical importance, they serve as an adequate benchmark for the purpose of validation. As the first validation example, the difference in ligand dissociation energies between Cr­(CO)_5_ and Pd­(PH_3_)_3_ fragments (denoted as Δ*E*) was selected as the objective value, and ligands that reduce the Δ*E* values were sought. First, the VLAO calculation was performed to obtain the optimal VL parameter p* (Figure 7). Instead of using the Δ*E* value itself as an objective function (represented by the crosses in the plot), the Δ*E* value corrected by the barrier function *B*(**p**) was minimized (the circles). This barrier function constrains the VL parameter **p** to a “realistic” range (see the function denoted as *P* in the previous work^23^ for details), preventing the optimal parameter from deviating excessively from those of real ligands (Table 1). In this study, the parameter ranges were set as follows: 1.3–1.9 Å for *r*_0_, 2.0–3.5 Å for *a*_1_, and 1.5–3.0 Å for *b*_1_. The impact of these range settings on prediction results is discussed in Subsection [Sec sec3.6]. Starting from the same initial parameters as the VLAO calculation in Subsection [Sec sec3.1], the optimal parameter set p* = (*r*_0_,*a*_1_,*b*_1_) = (1.35 Å, 2.13 Å, 1.64 Å) was obtained after 41 iterations, reducing the Δ*E* value from −22.1 kcal/mol to −32.4 kcal/mol. Next, the gradient and Hessian matrix of the uncorrected Δ*E* in terms of the VL parameter **p**, namely (dΔ*E*/d**p**)­(p*) and (d^2^Δ*E*/d**p** ^2^)­(p*), were calculated. Based on these, the function ${\mathrm{F̃}}_{virt}$ was defined as the second order Taylor expansion. It should be noted that the optimal parameter set p* corresponds to a local minimum of Δ*E* + *B*(**p**), rather than that of Δ*E* alone. Therefore, the gradient may have nonnegligible components, and the Hessian can possess negative eigenvalues. Indeed, in the present case, the gradient with respect to the electronic parameter *r*_0_ was found to be significantly positive, and the Hessian had a negative eigenvalue (see Figure S1). Since negative eigenvalues in the Hessian can cause a significant decrease in ${\mathrm{F̃}}_{virt}\left(p\right)$ when **p** deviates substantially from p*, which is apparently unreasonable, we clipped them to ensure the Hessian is positive semidefinite.

**Figure 7:**
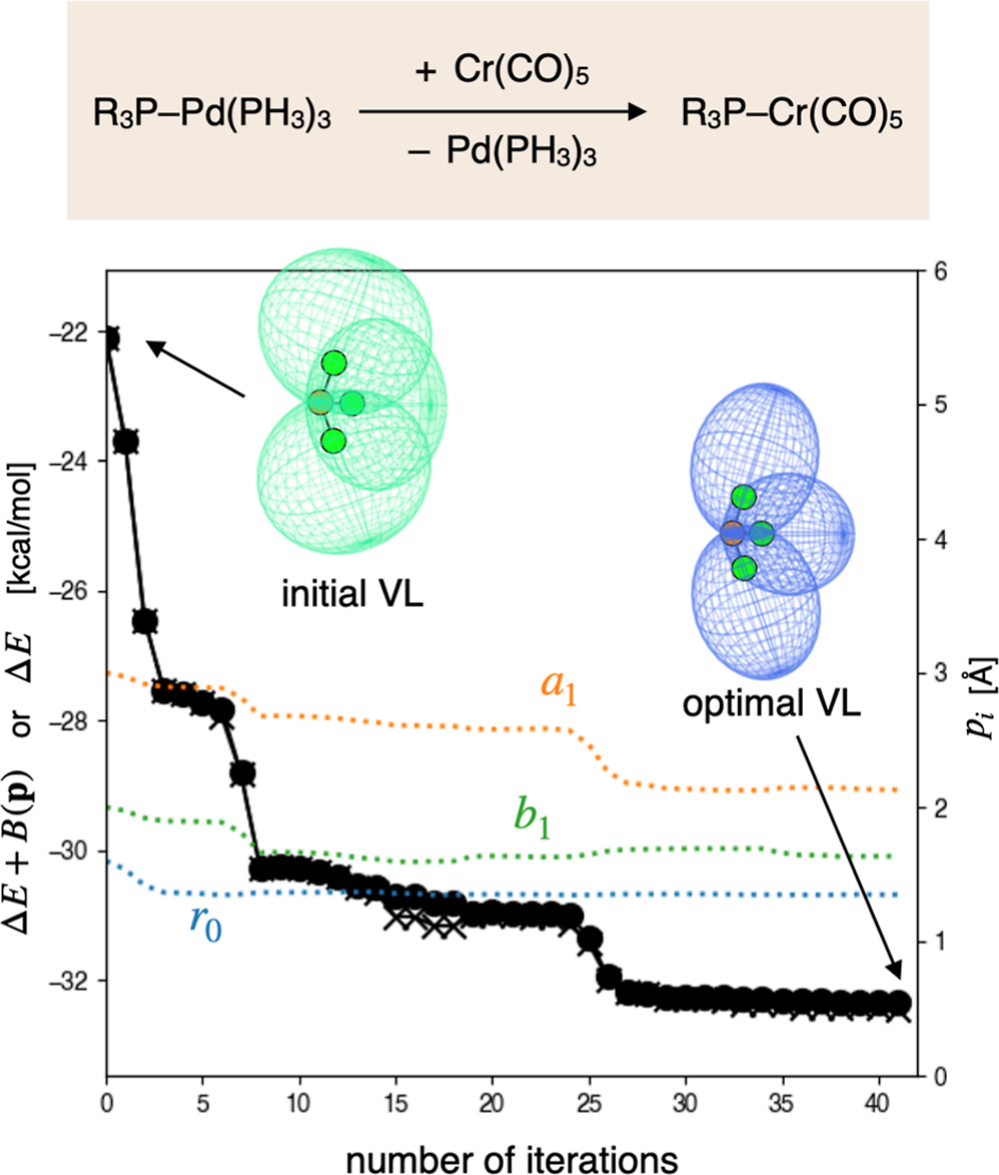
VLAO calculation to minimize Δ*E* + *B*(**p**). The VL parameters are visualized by the size, shape, and color of the ovoids.

With the function ${\mathrm{F̃}}_{virt}$ defined, the Δ*E* values of 21 ligands in the database were predicted by ${\mathrm{F̃}}_{virt}\left(H\left(G_{real}\left(R\right)\right)\right)$ , and plotted against *F*_real_(*R*) (Figure 8a). In this plot, circles represent the results for the 21 real ligands. The outcome of the VLAO calculation, in which the Δ*E* value of the VL was reduced from −22.1 kcal/mol to −32.4 kcal/mol, is depicted as a black square and a yellow star connected by an arrow. The star also corresponds to the reference point of the Taylor expansion. The color of each circle indicates the residual distance between a real ligand and its corresponding VLs in the 16-dimensional descriptor space (i.e., *l*_min_ in Table 1). Since this distance reflects how well a real ligand is reproduced by the VL, it serves as an indicator of prediction accuracy: predictions for real ligands with small *l*_min_ values (blue) are more reliable than those with large *l*_min_ values (red). Additionally, the transparency of the circles represents the normalized deviation of the VL parameters ∥Δ**p** ^′^∥, which is defined as follows

$$∥\Delta p^{\prime }∥≔\sqrt{\sum_{i}{\left(\frac{p_{i}-p_{i}^{*}}{p_{i}^{\mathrm{Max}}-p_{i}^{\min}}\right)}^{2}}$$

where *p*_*i*_ and *p*_i_^*^ represent the *i*-th components of the VL parameter of the corresponding ligand (**p** in Table 1) and the reference point of the Taylor expansion (p*), respectively. In addition, *p*_*i*_^*Max*^ and *p*_*i*_^*min*^ are maximum and minimum accessible values of the *i*-th component of the VL parameter. These values were set to match the parameter range for the barrier function *B*(**p**) in the VLAO calculation. Since the accuracy of the Taylor expansion decreases due to anharmonicity as ∥Δ**p** ^′^∥ increases, this value also serves as a reliability metric: predictions for real ligands close to the reference point p* (shown by opaque circles) are more reliable, as ${\mathrm{F̃}}_{virt}$ would be a good approximation of *F*_virt_. Considering these indicators, the prediction of Δ*E* was quantitatively accurate, as most ligands—except for those with low reliability (represented by red and/or transparent circles), such as **L3**, **L5**, **L6**, **L8**, **L15**, and **L21**—closely aligned with the diagonal. To enable more detailed discussions, *l*_min_ was plotted against ∥Δ**p** ^′^∥ as shown in Figure 8b. In this plot, ligands closer to the origin have higher prediction reliability than those further away. When this prediction algorithm is employed in practice—where chemists attempt to identify real ligands with better *y* values (smaller Δ*E* values in this case) without knowing *F*_real_(*R*)—these indicators of prediction reliability would be very helpful for filtering out ligands with unreliable predictions. Thus, 21 ligands were filtered based on their *l*_min_ and ∥Δ**p** ^′^∥ values (below 2.0 and 0.75, respectively), as shown by the blue dotted lines in Figure 8b. The remaining six ligands were then plotted in Figure 8c using the same approach as Figure 8a. As a result, all ligands aligned closely with the diagonal, indicating that their Δ*E* values were predicted accurately. In practical applications, since *F*_real_(*R*) is unknown, experiments would likely be conducted first on these reliable ligands, prioritizing those with the lowest predicted Δ*E*. Assuming this, the ligand with the second smallest Δ*E* among the 21 candidates (**L7**) would be identified in the first experiment, highlighting the utility of this prediction algorithm. It should be noted that the thresholds for prediction reliability were arbitrarily determined, and these values can be adjusted depending on various factors. For example, if the time and material costs per experiment are not extremely high, thus allowing for an increase in the number of experiments, it is advisible to set looser thresholds in order to test more ligands with acceptable reliability. Indeed, if the thresholds were set to 2.5 for *l*_min_ and 1.0 for ∥Δ**p** ^′^∥ (indicated by the red dotted lines in Figure 8b), the best ligand (**L9**) among 21 ligands would be found in the third experiment as shown in the Figure 8d. Notably, even with these criteria, the prediction accuracy remained very high for the 12 ligands selected.

**Figure 8:**
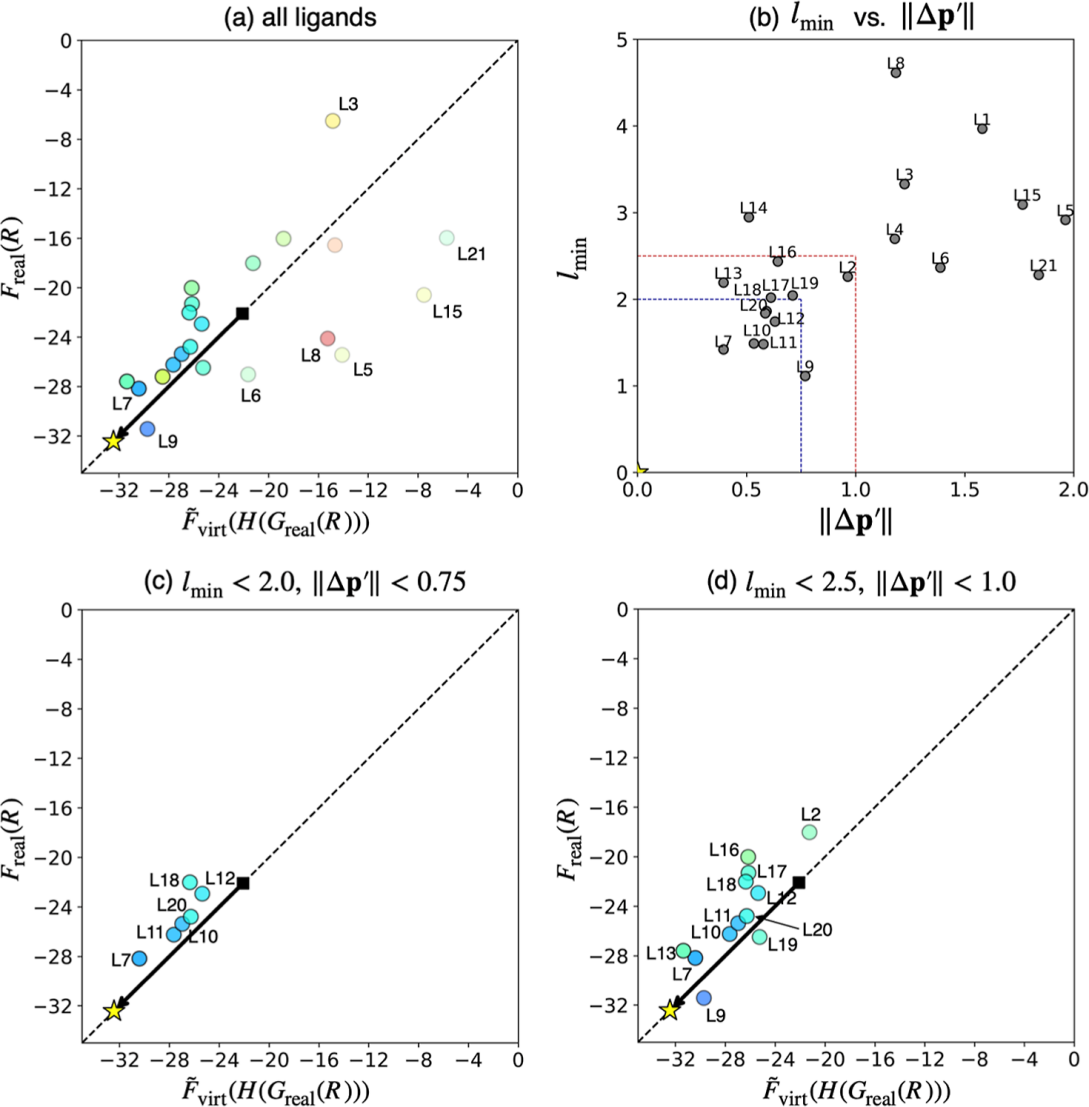
Internal validation of the prediction algorithm. The objective value was set as the difference in ligand dissociation energies (Δ*E*) between R_3_PCr­(CO)_5_ and R_3_PPd­(PH_3_)_3_. (a) Correlation between predicted and calculated reaction energies for all ligands. (b) Filtering criteria based on *l*_min_ and ∥Δ**p** ^′^∥ values. (c,d) Correlation between predicted and calculated reaction energies for the reliable ligands. In (a,c,d), *F*_real_(*R*) and ${\mathrm{F̃}}_{virt}\left(H\left(G_{real}\left(R\right)\right)\right)$ correspond to the calculated and predicted Δ*E* values in kcal/mol, respectively. Color and transparency of the circles represent *l*_min_ and ∥Δ**p** ^′^∥ values of the corresponding ligands. The star and the square represent Δ*E* value corresponding to the optimal and initial parameters of the VLAO calculation.

Another internal validation was performed using the same procedure. Figure 9 shows the result for a formal transmetalation of phosphine ligands from AgCl fragment to [PdCl_3_]^−^ fragment $\left(R_{3}PAgCl+{\left[{PdCl}_{3}\right]}^{-}\to AgCl+{\left[R_{3}P{PdCl}_{3}\right]}^{-}\right)$ . The prediction algorithm was again applied to identify ligands with low Δ*E* values, and the VLAO calculation was performed to minimize Δ*E* + *B*(**p**) (see Figure S3 for the results of the VLAO calculation). ${\mathrm{F̃}}_{virt}$ was then determined by the Taylor expansion around the resulting parameter p*. Finally, the Δ*E* values were predicted by evaluating ${\mathrm{F̃}}_{virt}\left(H\left(G_{real}\left(R\right)\right)\right)$ and the predicted values were compared to the calculated values (*F*_real_(*R*)) using the corresponding real ligands (Figure 9a). Again, the predictions were quantitatively accurate for the “reliable” ligands, indicated by the blue and opaque circles. Such ligands were systematically specified using the same criteria as in the previous example, resulting in the selection of three ligands for the strict thresholds and 11 ligands for the loose threshold (Figure 9b and c). With the loose criteria (Figure 9c), the second-best ligand (**L2**) among 21 ligands would be identified in the first experiment, demonstrating the utility of this prediction algorithm. In contrast, with the strict criteria (Figure 9b), **L11**, which ranks ninth among 21 ligands, would be identified as the best ligand. Although this may seem disappointing, **L11** shows better performance (i.e., the smaller Δ*E* value) than the value predicted by the VL (indicated by the star). Hence, assuming that the performance of the VL has reached a sufficient level through the VLAO calculation (otherwise, the post-VLAO process would not be performed), this result can be considered as the prediction algorithm successfully identified the most reliable ligand with a sufficient performance as the first choice. Note that the prediction algorithm filtered out **L1**, the best ligand among 21 ligands, due to its large ∥Δ**p** ^′^∥ and *l*_min_ values (see Figure S4b). Furthermore, even without filtering (i.e., assuming that experiments were performed based on Figure 9a), **L1** would be the 16th ligand to be tested due to its substantial prediction error. This example highlights two major limitations of the current prediction algorithm: First, since the optimal parameter p* obtained from the VLAO calculation is only a local maximum/minimum, the large ∥Δ**p** ^′^∥ value could cause a significant prediction error, not only due to the limited accuracy of the Taylor expansion, but also due to the potential overlooking of other local or global maxima/minima. Second, when the *l*_min_ value is large, the prediction becomes less reliable due to the deviation between the real ligand and its corresponding VL in the direction orthogonal to the *G*_virt_(*P*) surface (see Figure 3). Since the VLAO calculation is equivalent to finding one point on the *G*_virt_(*P*) surface that maximizes/minimizes an objective value *y*, the effect of the deviation orthogonal to the surface cannot be discussed in the current framework. Potential solutions for these limitations will be discussed in Subsection [Sec sec3.5].

**Figure 9:**
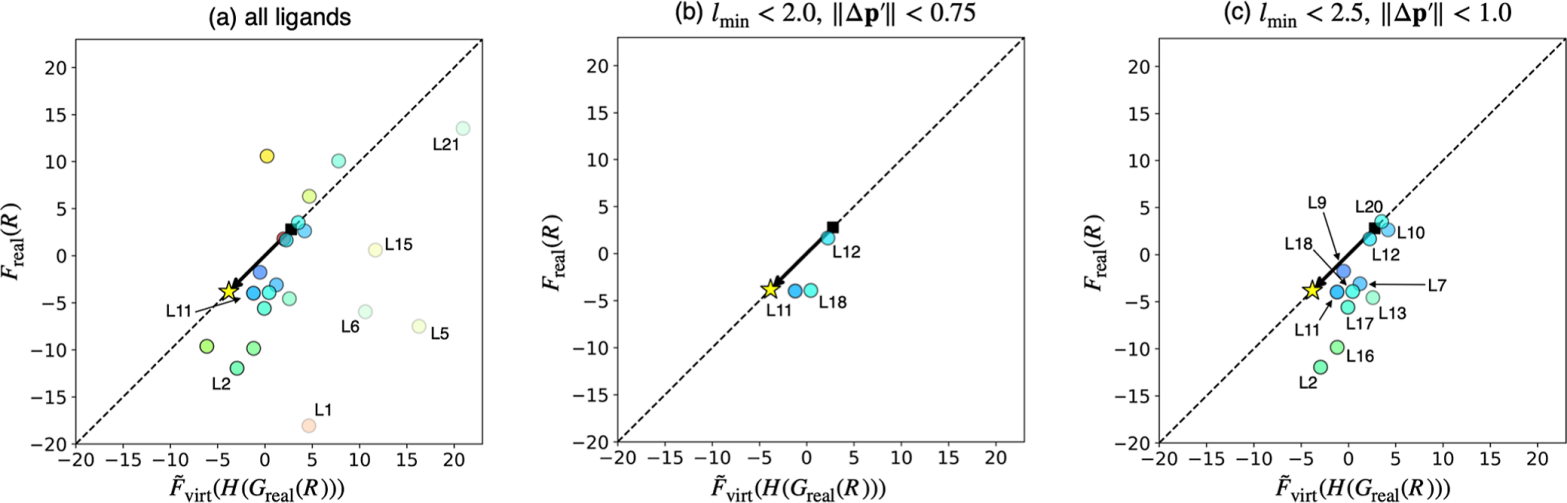
Internal validation of the prediction algorithm. The objective value was set as the difference in ligand dissociation energies (Δ*E*) between [R_3_PPdCl_3_]^−^ and R_3_PAgCl. (a) Correlation between predicted and calculated reaction energies for all ligands. (b,c) Correlation between predicted and calculated reaction energies for the reliable ligands. *F*_real_(*R*) and ${\mathrm{F̃}}_{virt}\left(H\left(G_{real}\left(R\right)\right)\right)$ correspond to the calculated and predicted Δ*E* values in kcal/mol, respectively. Color and transparency of the circles represent *l*_min_ and ∥Δ**p** ^′^∥ values of the corresponding ligands. The star and the square represent Δ*E* value corresponding to the optimal and initial parameters of the VLAO calculation.

### 3.3 External Validation 1

With the utility of the prediction algorithm clarified through internal validations, we conducted externals validation to assess its robustness. We first focused on the isomerization of *eq*-R_3_PRhH­(CO)_3_ to give *ax*-R_3_PRhH­(CO)_3_ (Figure 10). These rhodium complexes are key intermediates in hydroformylation, and the correlation between the isomeric ratio of this type of complex, particularly for bidentate phosphine complexes, and their catalytic activities has been widely investigated.^43–47^ Here, we aimed to control this energy difference using simple, monodentate phosphine ligands, guided by our prediction algorithm. As we had previously calculated the stability of several *eq*-R_3_PRhH­(CO)_3_ and *ax*-R_3_PRhH­(CO)_3_ complexes, we conducted a quasi-blind test. The procedure used in the internal validation was applied without modifications to minimize influence of prior knowledge and unconscious bias. Figure 10a shows a part of the results, where the VLAO calculation was performed to minimize Δ*E* + *B*(**p**). This calculation aims to identify real ligands with lower Δ*E* values, and the optimal parameter set p* was determined to be (*r*_0_,*a*_1_,*b*_1_) = (1.35 Å, 3.01 Å, 1.99 Å). Compared to the initial parameter set ((*r*_0_,*a*_1_,*b*_1_) = (1.6 Å, 3.0 Å, 2.0 Å)), *r*_0_ differs significantly, while *a*_1_ and *b*_1_ remain almost unchanged (see Figure S5 for details). This indicates that the energy difference is strongly influenced by the electronic effects. The function ${\mathrm{F̃}}_{virt}$ was then determined by the Taylor expansion around p*, and the Δ*E* values for all 21 ligands were predicted by evaluating ${\mathrm{F̃}}_{virt}\left(H\left(G_{real}\left(R\right)\right)\right)$ . “Reliable” sets of ligands were identified by filtering off ligands that did not satisfy the same criteria used in the internal validation. Under the “strict” criteria (*l*_min_ < 2.0 and ∥Δ**p** ^′^∥<0.75) six ligands were selected, while under the “loose” criteria (*l*_min_ < 2.5 and ∥Δ**p** ^′^∥<1.0), 12 ligands were obtained (see Figure S6b for details). At this stage, the predicted Δ*E* values of these ligands were compared with those calculated using the corresponding real ligands (*F*_real_(*R*)) as shown in Figure 10a. To our delight, most ligands in plot (i) and plot (ii) aligned closely with the diagonal, demonstrating the quantitative accuracy of the predictions. Assuming *F*_real_(*R*) was completely unknown and that calculations using real ligands (or experiments) were conducted in order of increasing predicted Δ*E*, **L10**, the second-best among the 21 ligands, was identified in the first trial under the strict criteria (plot (i)), while **L13**, the best ligand, was specified in the second trial under the loose criteria (plot (ii)). These results demonstrate reliability of this method.

**Figure 10:**
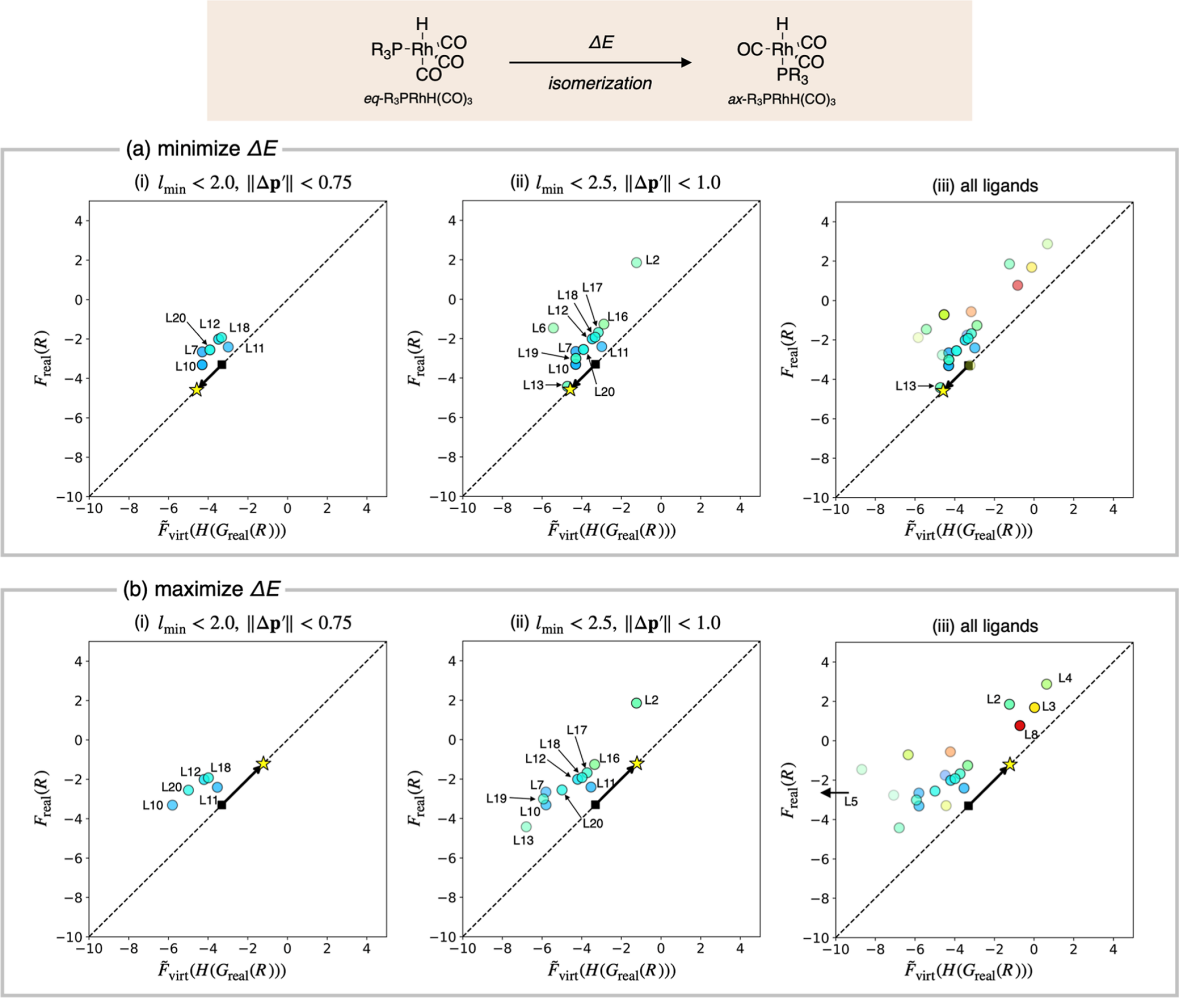
External validation of the prediction algorithm. The objective value was set as the energy difference (Δ*E*) between *eq*-R_3_PRhH­(CO)_3_ and *ax*-R_3_PRhH­(CO)_3_. (a) The attempt for finding ligands which minimize Δ*E*. (b) The attempt for finding ligands which maximize Δ*E*. *F*_real_(*R*) and ${\mathrm{F̃}}_{virt}\left(H\left(G_{real}\left(R\right)\right)\right)$ correspond to the calculated and predicted Δ*E* values in kcal/mol, respectively. Color and transparency of the circles represent *l*_min_ and ∥Δ**p** ^′^∥ values of the corresponding ligands. The star and the square represent Δ*E* value corresponding to the optimal and initial parameters of the VLAO calculation.

Figure 10b presents the predictions results where the VLAO calculation was performed to maximize Δ*E*-*B*(**p**), aiming to identify ligands with higher Δ*E* values. After the optimal parameter set p*was determined as (*r*_0_,*a*_1_,*b*_1_) = (1.85 Å, 2.99 Å, 2.01 Å), the Δ*E* values were predicted for all 21 ligands. Applying the filtration criteria resulted in five ligands under the strict criteria and 11 ligands under the loose criteria (see Figure S8b for details). The predicted Δ*E* values were then compared with calculated values (plot (i) and (ii)). Assuming that calculations using real ligands were conducted in order of decreasing predicted Δ*E*, **L18**, the twelfth-best ligand, was identified in the second trial under the strict criteria (plot (i)), while **L12**, the second-best ligand, was identified in the first trial under the loose criteria (plot (ii)). The former result seems disappointing, as **L18** exhibited only moderate performance and did not reach the level of the optimal VL. This result is due to an overly strict threshold, which led to the exclusion of four high performing but less reliable ligands (**L2**, **L3**, **L4** and **L8**). Since *l*_min_ remains constant regardless of the reaction (see Table 1), this outcome can be attributed to the large ∥Δ**p** ^′^∥, indicating that the VL parameter converged to a region in the descriptor space far from the real ligands (compare Figures S6b and S8b). As shown in plot (ii), relaxing the threshold enabled the discovery of **L2**. These results suggest that, depending on the outcome of the VLAO calculation, filtering based on ∥Δ**p** ^′^∥ could limit the discovery of better ligands. Possible solutions to this limitation are discussed in Subsections [Sec sec3.5] and 3.6. Overall, at least under the loose filtering criteria, the prediction algorithm successfully identified both the ligand with the lowest Δ*E* (**L13**) and the ligand with the second highest Δ*E* (**L2**) in a single trial each. These ligands have calculated Δ*E* values of −4.4 and 1.9 kcal/mol, respectively, and are therefore expected to reverse the equilibrium ratio of the *eq*-R_3_PRhH­(CO)_3_ and *ax*-R_3_PRhH­(CO)_3_ complexes.

### 3.4 External Validation 2

Another external validation was conducted, focusing on the C–H activation reaction of arylpalladium­(II) complex **A** through the concerted metalation-deprotonation (CMD) mechanism (Scheme 1).^48–51^ Since this elementary reaction step is important for Pd-catalyzed transformations of inert C–H bonds, ligands that lower the activation barrier could facilitate various C–H activation reactions under mild reaction conditions. Furthermore, because the activation barrier is sensitive to the electronic and steric effects of phosphine ligands,^20^ this reaction serves as a suitable model for validation. Following the same procedure as in the previous examples, the VLAO calculation was performed to minimize the activation barrier Δ*E* ^‡^ (see Figure S9 for details), leading to the convergence of the VL parameter at p* = (*r*_0_,*a*_1_,*b*_1_) = (1.35 Å, 2.90 Å, 1.62 Å), which resulted in a sufficiently low activation energy (25.5 kcal/mol). The derivative values at the optimal point p* were calculated, ${\mathrm{F̃}}_{virt}$ was determined by the Taylor expansion, and the Δ*E* ^‡^ values for all 21 ligands were then predicted. Filtration based on *l*_min_ and ∥Δ**p** ^′^∥ gave six ligands under the strict criteria and 13 ligands under the loose criteria (see Figure S10b for details). The predicted Δ*E* ^‡^ values were then compared with the calculated values (Figure 11). Again, assuming that calculations using real ligands were performed in order of increasing predicted Δ*E* ^‡^, **L7** and **L6**—ranked fourth and second among the 21 ligands—were identified in the first trial under the strict and loose criteria, respectively (Figure 11a,b).

**Scheme 1:**
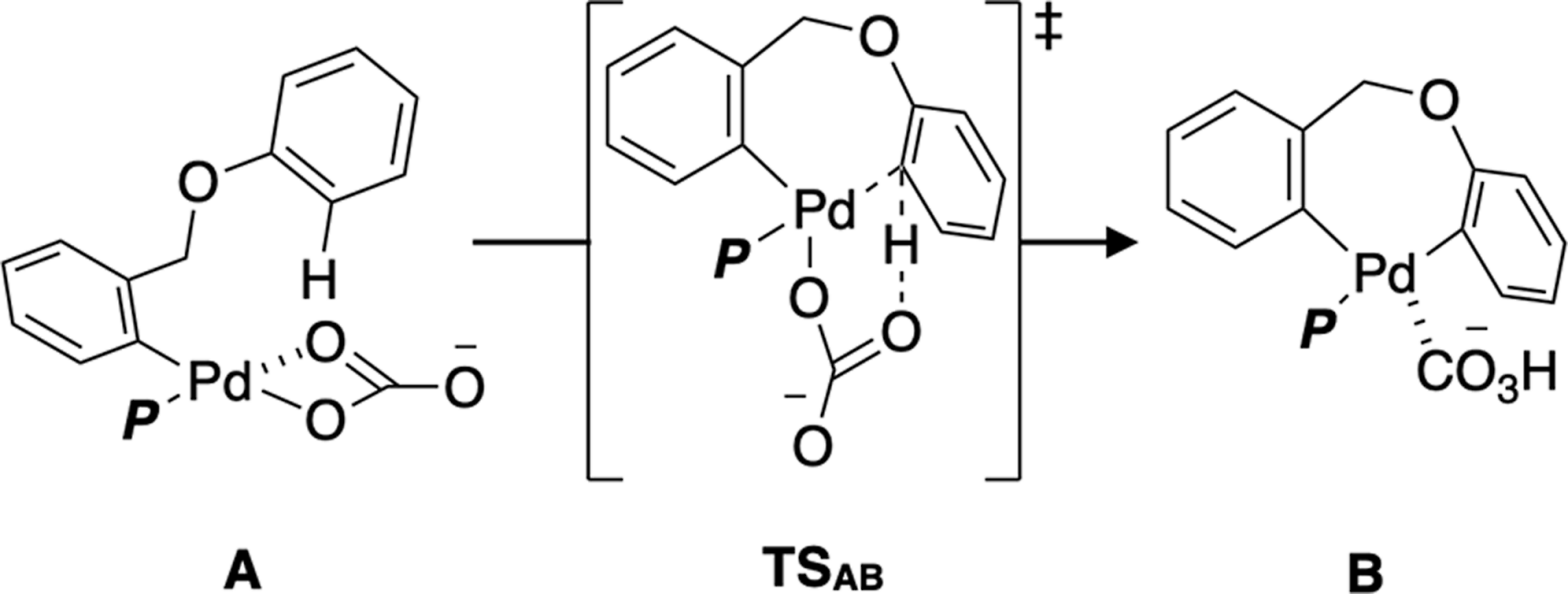
C–H Activation Reaction on the Pd­(II) Complex **A** through CMD Mechanism

**Figure 11:**
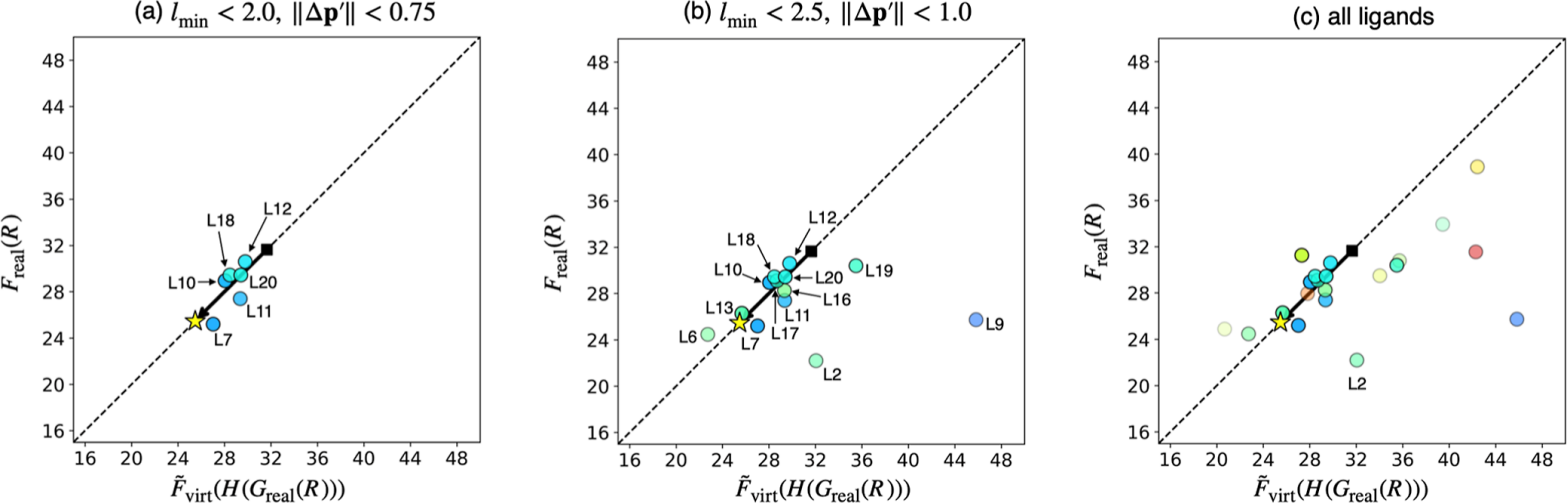
External validation of the prediction algorithm. The objective value was set as the activation energy (Δ*E* ^‡^) of the C–H activation reaction on the Pd­(II) complex **A**. (a,b) Correlation between predicted and calculated activation energies for the reliable ligands. (c) Correlation between predicted and calculated activation energies for all ligands. *F*_real_(*R*) and ${\mathrm{F̃}}_{virt}\left(H\left(G_{real}\left(R\right)\right)\right)$ correspond to the calculated and predicted Δ*E* ^‡^ values in kcal/mol, respectively. Color and transparency of the circles represent *l*_min_ and ∥Δ**p** ^′^∥ values of the corresponding ligands. The star and the square represent Δ*E* ^⧧^ value corresponding to the optimal and initial parameters of the VLAO calculation.

Although the algorithm successfully identified real ligands with sufficiently small Δ*E* ^⧧^ values in a minimum number of trials, it is important to note that in Figure 11b, the predicted Δ*E* ^⧧^ values of two ligands—**L2** and **L9**—deviated significantly from calculated values, despite the application of filtering to enhance prediction reliability. A detailed analysis revealed that, these discrepancies stem from two factors. The first is the overestimation of Δ*E* ^⧧^ due to large Hessian components, which accounts for the error observed in **L9**. At the reference point of the Taylor expansion, the second-order derivative of *F*_virt_ with respect to *r*_0_ and *b*_1_ has a significantly large (11.7 and 30.3 kcal/mol·Å^2^, respectively; see Figure S9). As mentioned earlier, the accuracy of ${\mathrm{F̃}}_{virt}$ decreases due to the anharmonicity of *F*_virt_, and this effect is expected to be more significant when the original curvature is larger. To evaluate the impact of this effect, ${\mathrm{F̃}}_{virt}$ was compared to *F*_virt_ as shown in the Figure 12a. In this plot, the orange line and blue dots represent the values of ${\mathrm{F̃}}_{virt}$ and *F*_virt_ with respect to changes in *b*_1_ parameter, respectively, while the star marks the reference point of the Taylor expansion. The other VL parameters, namely *r*_0_ and *a*_1_, were fixed to be the same as in p*. As the absolute value of Δ*b*_1_ = *b*_1_-*b*_1_^*^ increases, the discrepancy between ${\mathrm{F̃}}_{virt}$ and *F*_virt_ becomes larger, and at Δ*b*_1_ = ± 1.0 Å, the error reaches approximately 10 kcal/mol. Considering large Δ*b*_1_ value of **L9** (−1.01 Å), along with nonnegligible deviations in *r*_0_ and *a*_1_, the large error for **L9** would be reasonable. The second factor contributing to the discrepancies is the presence of strong attractive interactions between ligands and substrates, which accounts for the error observed in **L2**.^52–54^ The IGMH analysis^55–58^ of **TS**_**AB**_ bearing tris­(pentafluorophenyl)­phosphine (**L2**) was performed and visualized in Figure 12b. This analysis revealed an interaction between the negatively charged carbonate anion and the positively polarized C_6_F_5_ ring, which likely accounts for the unexpectedly small activation barrier of **L2**. Since such functional group-specific interactions, including ionic, π–π and CH/π interactions, cannot be described by the current VL model, neglecting these important interactions which may enhance ligand performance could be a limitation of this approach. Potential solutions to these limitations will be discussed from the next subsection.

**Figure 12:**
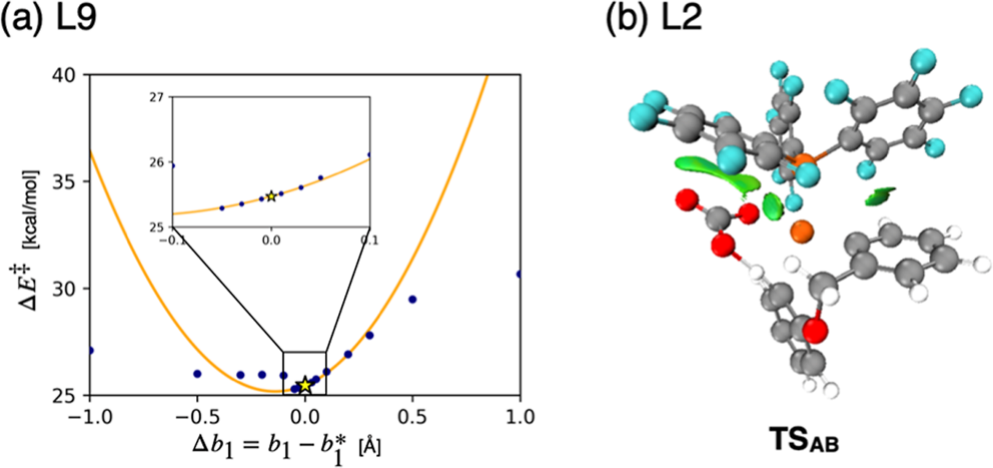
Two factors contributing to the prediction errors in Figure 11b. (a) The discrepancy between *F*_virt_ and ${\mathrm{F̃}}_{virt}$ due to the large curvature of *F*_virt_. (b) Interaction between carbonate anion and C_6_F_5_ ring in **L2**.

### 3.5 Limitations and Potential Improvements

In the previous discussion, we found four limitations of the present algorithm: (1) the possibility of VL parameters converging to an “unrealistic” region, where no real ligands are in proximity (e.g., Figure 10b); (2) the limited accuracy of ${\mathrm{F̃}}_{virt}$ as an approximation of *F*_virt_ (e.g., **L9** in Figure 11b); (3) the reduced reliability of ${\mathrm{F̃}}_{virt}\left(H\left(G_{real}\left(i\right)\right)\right)$ as *l*_min_ increases (e.g., **L1** in Figure 9); and (4) the limited reliability of ${\mathrm{F̃}}_{virt}\left(H\left(G_{real}\left(i\right)\right)\right)$ when functional group-specific interactions between ligands and substrates are significant (e.g., **L2** in Figure 11b). A straightforward approach to address the limitation (1) is to increase the number of ligands in the database, ensuring that the descriptor space is more densely and broadly populated. In this regard, linking with large-scale databases—such as LKB, kraken, tmQMg-L, and others^36–40,59–63^ —would be of considerable interest. We are also developing our own extended database, which includes not only monodentate phosphines but also other classes of ligands, thereby broadening the applicability of the present method. Alternatively, introducing a more sophisticated barrier function *B*(**p**), instead of the current simple definition,^23^ could help VL parameters to converge closer to real ligands, thereby mitigating this issue. Limitation (2) could potentially be addressed by refining the definition of ${\mathrm{F̃}}_{virt}$ to ensure that the approximation holds globally. One simple approach is to introduce additional reference points for the Taylor expansion and define ${\mathrm{F̃}}_{virt}$ as a weighted average of the predicated performance based on each reference point. Instead of introducing ${\mathrm{F̃}}_{virt}$ , it may also be possible to directly calculate *F*_virt_ for all candidates, depending on the size of the ligand database. These potential improvements are further discussed in the next subsection. To address limitation (3), a potential approach is to introduce additional parameters into the VL, thereby increasing the dimensionality of the *G*_virt_(*P*) surface and consequently reducing *l*_min_ values. In this study, many parameters in the VL, such as ϕ_0_ for the keep angle potential, *c*_1_, *c*_2_ and *d* for the ovoid LJ potential, were kept constants for simplicity. Relaxing these parameters would help reduce *l*_min_ values. Limitation (4), which appears to be the most challenging to address, could potentially be mitigated by incorporating additional terms to account for functional group-specific interactions within the approximation scheme of the VL. For instance, attractive interactions such as hydrogen bonds, π–π interactions and CH/π interactions could potentially be approximated using a strategy similar to that of the ovoid LJ potential. This would involve adjusting the interaction strength, modifying the powers in the LJ potential, and appropriately selecting target atoms to accurately describe these interactions. We are currently working on resolving these issues.

### 3.6 Further Considerations on Model Functions to Approximate *F*_virt_

In this study, we used ${\mathrm{F̃}}_{virt}$ , or the second-order Taylor expansion of *F*_virt_, to approximate *F*_virt_. This function was proved to be useful, as it allows for the estimation of ligand performance across the entire database with only a single VLAO calculation. However, it was also found to introduce the limitations (1) and (2) described in the previous subsection. Specifically, the accuracy of ${\mathrm{F̃}}_{virt}$ as an approximation of *F*_virt_ decreases as the distance between the reference point p* and the VL parameters corresponding to a given real ligand increases. Therefore, it was crucial to carefully define *B*(**p**) and its parameter ranges to ensure that the VLAO calculations would converge near the real ligands. In this subsection, we discuss an alternative model function to approximate *F*_virt_ that could potentially mitigate this issue based on the example of the C–H activation reaction on the palladium center (Figure 11).

First, to assess the impact of the parameter range of *B*(**p**) on prediction accuracy, we re-executed the VLAO calculation to minimize Δ*E* ^⧧^ + *B*(**p**) with an expanded parameter range (Figure S11). This time, the ranges were set as 1.0–2.2 Å for *r*_0_, 1.5–5.5 Å for *a*_1_, and 0.5–2.5 Å for *b*_1_ to fully encompass all 21 real ligands. As a result, the optimal parameter set p* = (*r*_0_,*a*_1_,*b*_1_) = (1.09 Å, 2.85 Å, 1.29 Å) was obtained, leading to a reduction of the activation barrier to 23.6 kcal/mol. Based on the obtained parameters, we redefined ${\mathrm{F̃}}_{virt}$ following the same procedure as before, and the Δ*E* ^⧧^ values for 21 ligands were predicted (Figure 13a). Compared to the original results (Figure 11c), the Δ*E* ^⧧^ value of the optimal VL (indicated by the yellow star) decreased by approximately 2 kcal/mol, and was predicted to be lower than that of any real ligand. In addition, the predicted Δ*E* ^⧧^ values for all real ligands deviate significantly from the computed values. These suggest that expanding the accessible parameter range allowed the VL to converge to a more favorable parameter set for the reaction, while simultaneously increasing the distance between the real ligands and the reference point of the Taylor expansion. To address this issue, we then defined another function ${\mathrm{F̂}}_{virt}$ as the approximation of *F*_virt_ as follows

$${\mathrm{F̂}}_{virt}\left(p\right)={\left(\sum_{j}\frac{1}{∥\Delta p_{j}^{\prime }|{|}^{2}}\right)}^{-1}\sum_{i}\frac{{\mathrm{F̃}}_{virt}^{i}\left(p\right)}{∥\Delta p_{i}^{\prime }|{|}^{2}}$$

where the summation is taken over all iterations of the VLAO calculation. ${\mathrm{F̃}}_{virt}^{i}\left(p\right)$ is the approximation of *F*_virt_(**p**) calculated based on the second-order Taylor expansion around the parameter set **p**_*i*_ corresponding to *i*-th iteration. Therefore, ${\mathrm{F̂}}_{virt}\left(p\right)$ is the average of ${\mathrm{F̃}}_{virt}^{i}\left(p\right)$ values from each step of the VLAO calculation, weighted by the inverse square of distance ∥Δ**p**_*i*_^′^∥ between the real molecule and **p**_*i*_. As this function incorporates information not only from the end point of the VLAO calculation but also from the trajectory, *F*_virt_(**p**) can be accurately approximated as long as **p** is close to the trajectory in the parameter space. The Δ*E* ^‡^ values for all real ligands were predicted by calculating ${\mathrm{F̂}}_{virt}\left(H\left(G_{real}\left(R\right)\right)\right)$ , and the results are shown in Figure 13b. Compared to Figure 13a, which only uses information from the end point of the VLAO calculation, the prediction accuracy has significantly improved. Notably, as with ${\mathrm{F̃}}_{virt}\left(p\right)$ , ${\mathrm{F̂}}_{virt}\left(p\right)$ can be computed using only the results from the VLAO calculation, enabling predictions across all ligands in the database without requiring additional quantum chemical calculations. Thus, ${\mathrm{F̂}}_{virt}$ would be a promising alternative to ${\mathrm{F̃}}_{virt}$ and has the potential to resolve the limitations associated with ${\mathrm{F̃}}_{virt}$ . The prediction results for isomerization of the rhodium complexes (i.e., Subsection [Sec sec3.3]) based on ${\mathrm{F̂}}_{virt}$ are provided in the Supporting Information.

**Figure 13:**
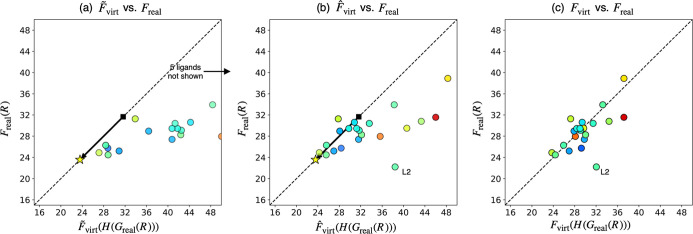
Comparison of model functions to approximate *F*_virt_. The objective value was set as the activation energy (Δ*E* ^⧧^) of the C–H activation reaction on the Pd­(II) complex **A**. *F*_real_(*R*) corresponds to the calculated Δ*E* ^⧧^ values in kcal/mol. ${\mathrm{F̃}}_{virt}\left(H\left(G_{real}\left(R\right)\right)\right)$ , ${\mathrm{F̂}}_{virt}\left(H\left(G_{real}\left(R\right)\right)\right)$ , *F*_virt_(*H*(*G*_real_(*R*))) correspond to the predicted Δ*E* ^⧧^ values in kcal/mol. Color and transparency of the circles represent *l*_min_ and ∥Δ**p** ^′^∥ values of the corresponding ligands. The star and the square represent Δ*E* ^‡^ value corresponding to the optimal and initial parameters of the VLAO calculation.

Finally, instead of using an approximate function for *F*_virt_, we used *F*_virt_(**p**) itself for the predictions. The Δ*E* ^⧧^ values for all real ligands were predicted by calculating *F*_virt_(*H*(*G*_real_(*R*))), and the results are shown in Figure 13c. Apparently, compared to Figures 11c, 13a,b, the prediction accuracy has dramatically improved, with the Δ*E* ^‡^ values quantitatively predicted for all ligands except **L2**. While VLAO calculations are not required for the calculation of *F*_virt_(*H*(*G*_real_(*R*))), quantum chemical calculations of the corresponding TS and EQ (**TS**_**AB**_ and **A**) are required in proportion to the number of real ligands, as mentioned in Subsection [Sec sec2.4]. Therefore, *F*_virt_(*H*(*G*_real_(*R*))) cannot be calculated when the number of real ligands in the database becomes very large. However, this can serve as a highly effective approach when considering a limited number of ligands, such as a few dozen in this study, or when the number of ligands is effectively reduced through prefiltering from a database.

### 3.7 Comparison with Established Prediction Methods

Given that the present algorithm differs fundamentally from conventional prediction approaches, such as quantitative structure–activity relationship (QSAR) models and machine learning methods, it is important to clarify its advantages and limitations. One of the key advantages of the present method is its independence from training data. Unlike conventional data-driven approaches, our framework is based entirely on quantum chemical calculations. This allows for performance prediction even in the absence of training data. For example, we attempted to construct prediction models for the activation energy of the CMD process (Scheme 1) using Lasso regression, Ridge regression, and random forest regression, but none yielded statistically meaningful results under leave-one-out cross-validation (see Figure S17 for the details). This is presumably due to the limited number of training samples (only 21 data points). In contrast, our method makes no use of such data in the prediction process, demonstrating its potential utility in data-scarce domains.

Despite its advantages, the present method has two notable limitations when compared to conventional prediction techniques. First, it requires a higher computational cost compared to purely data-driven models. While the present approach is designed to minimize computational costs, and the evaluation of ${\mathrm{F̃}}_{virt}$ requires only simple matrix operations with a computational cost scaling linearly with the number of database samples, the definition of ${\mathrm{F̃}}_{virt}$ itself involves iterative quantum chemical calculations (i.e., the VLAO calculation), which constitutes the main computational bottleneck of the method. The total number of iterations varies depending on the number of parameters, the characteristics of the objective function, and the efficiency of the line search. In the cases described in this study, the parameter optimization required up to approximately 150 iterations, including line search steps. It is worth noting, however, that the computational demand is significantly reduced compared to typical quantum chemical calculations, owing to the use of a small model ligand (PCl_3_) in place of computationally demanding real ligands, and the fact that each geometry optimization during the VLAO calculation is initialized from the previously converged structure, typically resulting in convergence within a few geometry updates. The second limitation is that the method assumes prior knowledge of the key elementary step responsible for catalytic performance. In cases where competing side reactions play a role, predictions based on a few limited reaction pathways may deviate from experimental observations. This issue could be addressed by integrating the method with automated reaction path search techniques such as the SC-AFIR method,^42^ a direction currently being explored in our ongoing research.

Taken together, the proposed approach represents a mechanism-based alternative to conventional data-driven prediction models. Its ability to operate without training data sets makes it particularly valuable for underexplored or novel catalytic systems. While it involves significant computational effort and requires mechanistic insights, these limitations are counterbalanced by its robustness and generalizability. While fundamentally different in nature, mechanism-based and data-driven approaches can be considered complementary, with each offering distinct advantages depending on the nature of the problem and data availability.

## 4 Conclusions

In this study, we proposed a mathematical approach to link VL parameters with real molecules, enabling the identification of optimal ligands based on the VLAO calculation. This algorithm evaluates the performance of real ligands in the target reaction through a three-step process: (1) calculating descriptor vectors using real molecules (denoted as *G*_real_), (2) converting the obtained descriptors into VL parameters (denoted as *H*), and (3) predicting ligand performance from the VL parameters (denoted as ${\mathrm{F̃}}_{virt}$ or ${\mathrm{F̂}}_{virt}$ ). Notably, since the functions *G*_real_ and *H* are independent of the target reaction, as demonstrated in our study, it is possible to create a parameter database in advance. If such a database is available, the performance of all ligands in the target reaction can be rapidly evaluated by performing a single VLAO calculation, without requiring additional quantum chemical calculations. Moreover, the algorithm provides not only predicted performance but also reliability indices (*l*_min_ and ∥Δ**p** ^′^∥), enabling the systematic selection of ligands to be tested. The filtering threshold can be adjusted based on various factors such as the required prediction accuracy and associated experimental costs. Both internal and external validations showed that the algorithm successfully identified ligands with sufficient performance. The limitations and potential improvements of the prediction algorithm were also discussed. Future efforts will be directed toward the continued improvement of the prediction algorithm.

## Supplementary Material



## Data Availability

Quantum chemical calculations were performed using the Gaussian 16 software^41^ and the GRRM23 software.^42^ The data, codes and input files used in this manuscript are provided at https://github.com/WatMat1127/V2R.

## Notes

Additional computational procedures, detailed data (PDF)
