# Supplementary material for: Mathematical Framework to Identify Optimal Molecule Based on Virtual Ligand Strategy
Source: J Chem Inf Model. 2025 Jun 13;65(13):6913–26. doi: 10.1021/acs.jcim.5c00815 (PMC12264936; doi:10.1021/acs.jcim.5c00815)
Supplement: Supplementary file 1 [file ci5c00815_si_001.pdf]

Supporting Information

Mathematical Framework to Identify Optimal Molecule based on Virtual Ligand Strategy

Wataru Matsuoka,*[a,b,c] Ken Hirose,[d] Ren Yamada,[d]
Taihei Oki,*[a,b] Satoru Iwata,[a,b,e] Satoshi Maeda*[a,b,c]

[a]*Institute for Chemical Reaction Design and Discovery (WPI-ICReDD), Hokkaido University, Kita 21, Nishi 10, Kita-ku, Sapporo, Hokkaido 001-0021, Japan.*

[b]*JST, ERATO Maeda Artificial Intelligence in Chemical Reaction Design and Discovery Project, Kita 10, Nishi 8, Kita-ku, Sapporo, Hokkaido 060-0810, Japan.*

[c]*Department of Chemistry, Faculty of Science, Hokkaido University, Kita 10, Nishi 8, Kita-ku, Sapporo, Hokkaido 060-0810, Japan.*

[d]*Graduate School of Chemical Science and Engineering, Hokkaido University, Kita 13, Nishi 8, Kita-ku, Sapporo, Hokkaido 060-8628, Japan.*

[e]*Department of Mathematical Informatics, Graduate School of Information Science and Technology, The University of Tokyo, Hongo 7-3-1, Bunkyo-ku, Tokyo 113-8656, Japan.*

e-mail addresses: matsuoka.wataru@sci.hokudai.ac.jp (W. Matsuoka)
oki@icredd.hokudai.ac.jp (T. Oki.)
smaeda@eis.hokudai.ac.jp (S. Maeda)

Table of Contents

1. General computational methods
2. Derivation of second derivatives
3. Detailed results of internal validations
4. Detailed results of external validations
5. Results with expanded parameter ranges
6. Additional internal validations
7. Attempts using conventional prediction methods
8. Supplemental references

1. General computational methods

All energy and gradient calculations at the density functional theory (DFT) level were performed using the Gaussian 16 software.[S1] Calculations at the GFN1-xTB level[S2] were performed using the ORCA 4.2.0 software.[S3] Geometry optimizations were conducted with the GRRM23 software.[S4] All DFT calculations were performed *in vacuo* using the ωB97X-D functional and the Def2-SVP basis set.

The energies of the transition metal complexes shown in Figure 6, *eq*-$R_3PRhH(CO)_3$ and *ax*-$R_3PRhH(CO)_3$ were determined based on the most stable conformer identified in the following conformation search. First, conformational isomers of the corresponding complexes were systematically explored using the single-component artificial force induced reaction (SC-AFIR) method[S4] at the GFN1-xTB level. All obtained equilibrium structures (EQs) were clustered based on their geometric similarity, and the most stable equilibrium geometry in each cluster was selected as the representative structure of that cluster. For representative structures that exhibit the same bond connectivity as the target structure, the relative energies from the most stable EQ among all were calculated. Then, structural optimizations at the ωB97X-D/Def2-SV(P) level were performed for all representative structures with relative energies below 5 kcal/mol. Based on the results, structural optimizations at the ωB97X-D/Def2-SVP level were performed for EQs with relative energies below 5 kcal/mol. If more than five such EQs were found, they were clustered into five groups based on geometric similarity, and only the most stable structure in each cluster was optimized. The energy of the complex was determined based on the most stable conformer obtained through this procedure. It should be noted that, for complexes such as *cis*-$R_3PRhCl(CO)_2$, *trans*-$R_3PRhCl(CO)_2$, *cis*-$R_3PIrCl(CO)_2$, *trans*-$R_3PIrCl(CO)_2$, *eq*-$R_3PRhH(CO)_3$ and *ax*-$R_3PRhH(CO)_3$, where geometrical isomers need to be distinguished, their geometries other than phosphine ligands were fixed during the conformation sampling using the SC-AFIR method.

The activation energy of the C–H activation reaction (Scheme 1) corresponding to a real ligand was calculated as follows. First, conformational flexibilities of the phosphine ligand in complex **A** and transition state **TS$_{AB}$** were systematically explored using the SC-AFIR method at the GFN1-xTB level, while keeping the geometries of all other components fixed. All obtained structures were clustered based on their geometric similarity, and the most stable geometry in each cluster was selected as the

representative structure of that cluster. For representative structures that exhibit the same bond connectivity as the input, the relative energies from the most stable EQ among all were calculated. Then, structural optimizations at the ωB97X-D/Def2-SVP level were performed for all representative structures with relative energies below 5 kcal/mol, while keeping the geometries other than the phosphine ligand fixed. Based on the results, all structures with relative energies below 2 kcal/mol were selected and all atoms in each structure were relaxed to a corresponding stationary point (EQ or TS). The activation energy was determined as the energy difference between the most stable conformer of **A** and $\mathbf{TS_{AB}}$.

All the VLAO calculations were performed as follows. VL parameters were optimized by the Conjugate-Gradient (CG) method based on the first-order derivative of an objective function F,. At each iteration, the VL parameters were updated based on the CG, followed by geometry optimizations of the corresponding TSs and EQs. The F value and its derivative ($\mathrm{d}F/\mathrm{d}\mathbf{p}$) were then evaluated. After each line search, the maximum absolute values among the gradient and displacement components were calculated, and convergence was determined when these values fell below the thresholds. The gradient threshold was set to 0.5 Å^{-1} for the optimization to minimize $l(\boldsymbol{x}, \mathbf{p})$ (subsection 3.1), and 1.0 kcal/(mol·Å)$^{-1}$ for the other optimizations minimizing or maximizing ΔE or $\Delta E^{\ddagger}$. The displacement threshold was set to 0.1 Å for all calculations. In all calculations, the optimal geometries for TSs and EQs, as well as the corresponding internal parameters for the VL ($\mathbf{q}$), from the previous step were used as the initial guesses for the next step.

2. Derivation of second derivatives

The second-order derivative of F_{virt} was derived as follows. The potential energy surface (PES) in the VL method is given by:

$$E(\mathbf{Q},\mathbf{p},\mathbf{q}) = E_{\mathrm{elec}}(\mathbf{Q}) + V_{\mathrm{VL}}(\mathbf{Q},\mathbf{p},\mathbf{q}),$$

where E_{elec} represents the electronic energy with respect to the atomic coordinate $\mathbf{Q}$, and V_{VL} is the penalty function. The penalty function consists of the sum of the keep potential, the keep angle potential and the ovoid LJ potential, which are determined by $\mathbf{Q}$, VL parameters $\mathbf{p}$ and internal parameters $\mathbf{q}$ in the ovoid LJ potential. When considering an EQ, as $\mathbf{Q}$ and $\mathbf{q}$ are optimized to corresponding minima, its energy can be expressed as follows:

$$E_{\mathrm{EQ}}(\mathbf{p}) = E\big(\mathbf{Q}^*(\mathbf{p}),\mathbf{p},\mathbf{q}^*(\mathbf{Q}^*(\mathbf{p}),\mathbf{p})\big),$$

where $\mathbf{q}^*(\mathbf{Q},\mathbf{p})$ represents the internal parameters that minimize $E(\mathbf{Q},\mathbf{p},\mathbf{q})$ for given $\mathbf{Q}$ and $\mathbf{p}$, *i.e.*,

$$\mathbf{q}^*(\mathbf{Q},\mathbf{p}) = \arg\min_{\mathbf{q}}[E(\mathbf{Q},\mathbf{p},\mathbf{q})],$$

and $\mathbf{Q}^*(\mathbf{p})$ is the local minimum of a function $\mathbf{Q} \mapsto E\big(\mathbf{Q},\mathbf{p},\mathbf{q}^*(\mathbf{Q},\mathbf{p})\big)$ within a neighborhood of the initial guess. Hereafter, we sometimes omit the arguments of functions for simplicity. As described in the previous work,[S5] the first-order derivative of E_{EQ} with respect to VL parameters $\mathbf{p}$ is given as

$$\frac{\mathrm{d}E_{\mathrm{EQ}}}{\mathrm{d}\mathbf{p}} = \frac{\partial V_{\mathrm{VL}}}{\partial \mathbf{p}}.$$

Hence, the second-order derivative can be expressed as follows:

$$\frac{\mathrm{d}^2E_{\mathrm{EQ}}}{\mathrm{d}\mathbf{p}^2} = \frac{\partial^2 V_{\mathrm{VL}}}{\partial\mathbf{p}\partial\mathbf{Q}}\frac{\mathrm{d}\mathbf{Q}^*}{\mathrm{d}\mathbf{p}} + \frac{\partial^2 V_{\mathrm{VL}}}{\partial\mathbf{p}^2} + \frac{\partial^2 V_{\mathrm{VL}}}{\partial\mathbf{p}\partial\mathbf{q}}\left(\frac{\partial\mathbf{q}^*}{\partial\mathbf{Q}}\frac{\mathrm{d}\mathbf{Q}^*}{\mathrm{d}\mathbf{p}} + \frac{\partial\mathbf{q}^*}{\partial\mathbf{p}}\right). \tag{1}$$

Since $\mathbf{Q}^*(\mathbf{p})$ is a local minimum of $\mathbf{Q} \mapsto E(\mathbf{Q},\mathbf{p},\mathbf{q}^*(\mathbf{Q},\mathbf{p}))$, it satisfies $\partial E/\partial\mathbf{Q} = 0$. Differentiating both sides of this equation with respect to $\mathbf{p}$ gives

$$\frac{\partial^2 E}{\partial\mathbf{Q}^2}\frac{\mathrm{d}\mathbf{Q}^*}{\mathrm{d}\mathbf{p}} + \frac{\partial^2 E}{\partial\mathbf{Q}\,\partial\mathbf{p}} + \frac{\partial^2 E}{\partial\mathbf{Q}\,\partial\mathbf{q}}\left(\frac{\partial\mathbf{q}^*}{\partial\mathbf{Q}}\frac{\mathrm{d}\mathbf{Q}^*}{\mathrm{d}\mathbf{p}} + \frac{\partial\mathbf{q}^*}{\partial\mathbf{p}}\right) = 0,$$

and rearranging this equation yields the following:

$$\begin{aligned}\frac{\partial\mathbf{Q}^*}{\partial\mathbf{p}} &= -\left(\frac{\partial^2 E}{\partial\mathbf{Q}^2} + \frac{\partial^2 E}{\partial\mathbf{Q}\,\partial\mathbf{q}}\frac{\partial\mathbf{q}^*}{\partial\mathbf{Q}}\right)^{-1}\left(\frac{\partial^2 E}{\partial\mathbf{Q}\,\partial\mathbf{p}} + \frac{\partial^2 E}{\partial\mathbf{Q}\,\partial\mathbf{q}}\frac{\partial\mathbf{q}^*}{\partial\mathbf{p}}\right)\\ &= -\left(\frac{\partial^2 E}{\partial\mathbf{Q}^2} + \frac{\partial^2 V_{\mathrm{VL}}}{\partial\mathbf{Q}\,\partial\mathbf{q}}\frac{\partial\mathbf{q}^*}{\partial\mathbf{Q}}\right)^{-1}\left(\frac{\partial^2 V_{\mathrm{VL}}}{\partial\mathbf{Q}\,\partial\mathbf{p}} + \frac{\partial^2 V_{\mathrm{VL}}}{\partial\mathbf{Q}\,\partial\mathbf{q}}\frac{\partial\mathbf{q}^*}{\partial\mathbf{p}}\right),\end{aligned} \tag{2}$$

where A^{-1} for a matrix A denotes the inverse matrix of A. Similarly, as $\mathbf{q}^*(\mathbf{Q},\mathbf{p})$ satisfies $\partial E/\partial\mathbf{q}\left(\mathbf{Q},\mathbf{p},\mathbf{q}^*(\mathbf{Q},\mathbf{p})\right)=0$, differentiating both sides of this equation by $\mathbf{Q}$ and $\mathbf{p}$ gives the following equations:

$$\frac{\partial\mathbf{q}^*}{\partial\mathbf{Q}}=-\left(\frac{\partial^2 V_{\mathrm{VL}}}{\partial\mathbf{q}^2}\right)^{-1}\left(\frac{\partial^2 V_{\mathrm{VL}}}{\partial\mathbf{q}\,\partial\mathbf{Q}}\right), \tag{3}$$

$$\frac{\partial\mathbf{q}^*}{\partial\mathbf{p}}=-\left(\frac{\partial^2 V_{\mathrm{VL}}}{\partial\mathbf{q}^2}\right)^{-1}\left(\frac{\partial^2 V_{\mathrm{VL}}}{\partial\mathbf{q}\,\partial\mathbf{p}}\right). \tag{4}$$

Using equations (1), (2), (3) and (4), the second-order derivative of E_{EQ} with respect to VL parameters $\mathbf{p}$ can be calculated. The second-order derivative values of E with respect to $\mathbf{Q}$ (*i.e.*, $\partial^2E/\partial\mathbf{Q}^2$) were obtained from the output file of the GRRM program. All the second-order derivative values of V_{VL} (*i.e.*, $\partial^2V_{\mathrm{VL}}/\partial\mathbf{p}\,\partial\mathbf{Q}$, $\partial^2V_{\mathrm{VL}}/\partial\mathbf{p}^2$, $\partial^2V_{\mathrm{VL}}/\partial\mathbf{p}\,\partial\mathbf{q}$, $\partial^2V_{\mathrm{VL}}/\partial\mathbf{Q}\,\partial\mathbf{q}$, and $\partial^2V_{\mathrm{VL}}/\partial\mathbf{q}^2$) were calculated using the automatic differentiation algorithm implemented in the PyTorch module[S6] for Python. The second-order derivative of E_{TS}, the energy of a transition state, with respect to VL parameters $\mathbf{p}$ can also be derived under the same discussion.

Assuming that F_{virt} is a function of energies corresponding to N stationary points (*i.e.*, EQ and TS), the first and second derivatives of F_{virt} with respect to $\mathbf{p}$ can be expressed as follows:

$$\frac{\mathrm{d}F_{\mathrm{virt}}}{\mathrm{d}\mathbf{p}}=\frac{\mathrm{d}F_{\mathrm{virt}}}{\mathrm{d}\mathbf{E}}\frac{\mathrm{d}\mathbf{E}}{\mathrm{d}\mathbf{p}},$$

$$\frac{\mathrm{d}^2F_{\mathrm{virt}}}{\mathrm{d}\mathbf{p}^2}=\left(\frac{\mathrm{d}\mathbf{E}}{\mathrm{d}\mathbf{p}}\right)^{\mathsf{T}}\frac{\mathrm{d}^2F_{\mathrm{virt}}}{\mathrm{d}\mathbf{E}^2}\frac{\mathrm{d}\mathbf{E}}{\mathrm{d}\mathbf{p}}+\frac{\mathrm{d}F_{\mathrm{virt}}}{\mathrm{d}\mathbf{E}}\frac{\mathrm{d}^2\mathbf{E}}{\mathrm{d}\mathbf{p}^2},$$

where A^{T} denotes the transpose of a matrix A, and $\mathbf{E}$ represents a vector consisting of the energies corresponding to the N stationary points (*i.e.*, $\mathbf{E}=(E_1,E_2,\ldots,E_N)^{\mathsf{T}}$).

3. Detailed results of internal validations

The detailed results of the internal validations were shown in Figure S1–S4. In the VLAO calculation, the parameter ranges of the barrier function $B(\mathbf{p})$ was set to 1.3–1.9 Å for r_0, 2.0–3.5 Å for a_1, and 1.5–3.0 Åfor b_1.

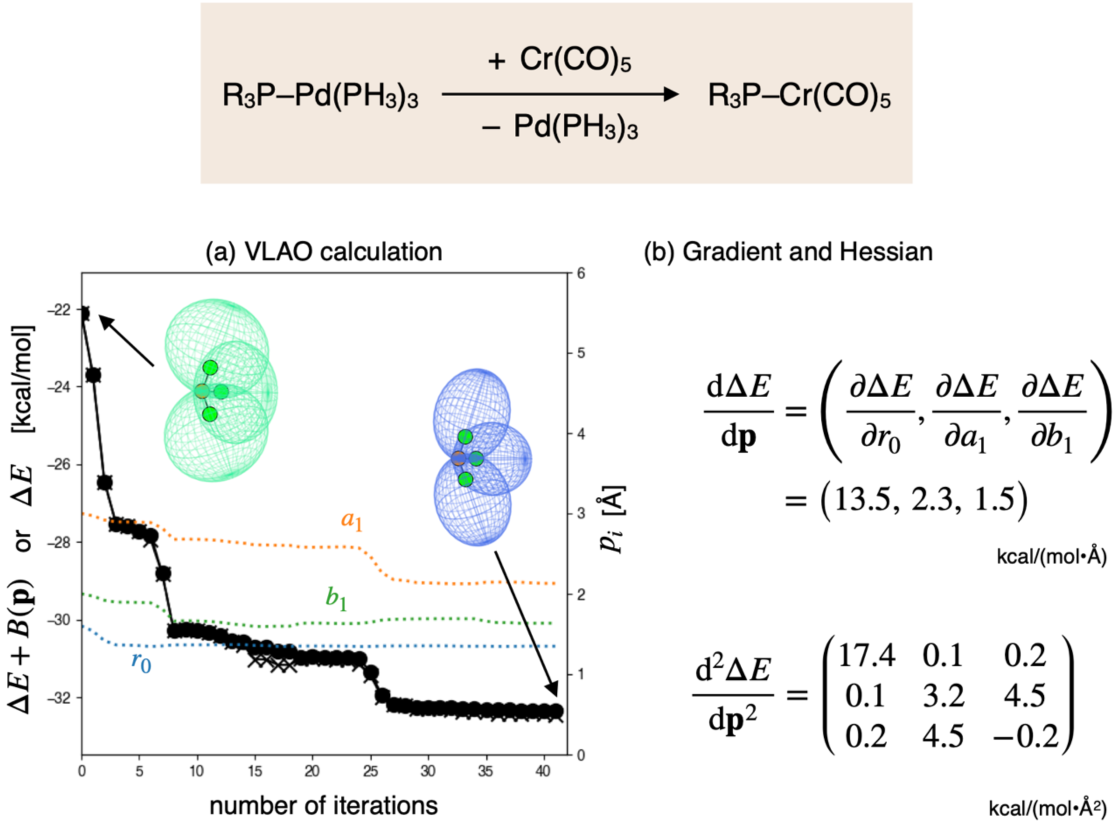

Figure S1. The detailed results of VLAO calculation to minimize $\Delta E + B(\mathbf{p})$, where ΔE represents the difference in ligand dissociation energies between $R_3PPd(PH_3)_3$ and $R_3PCr(CO)_5$.

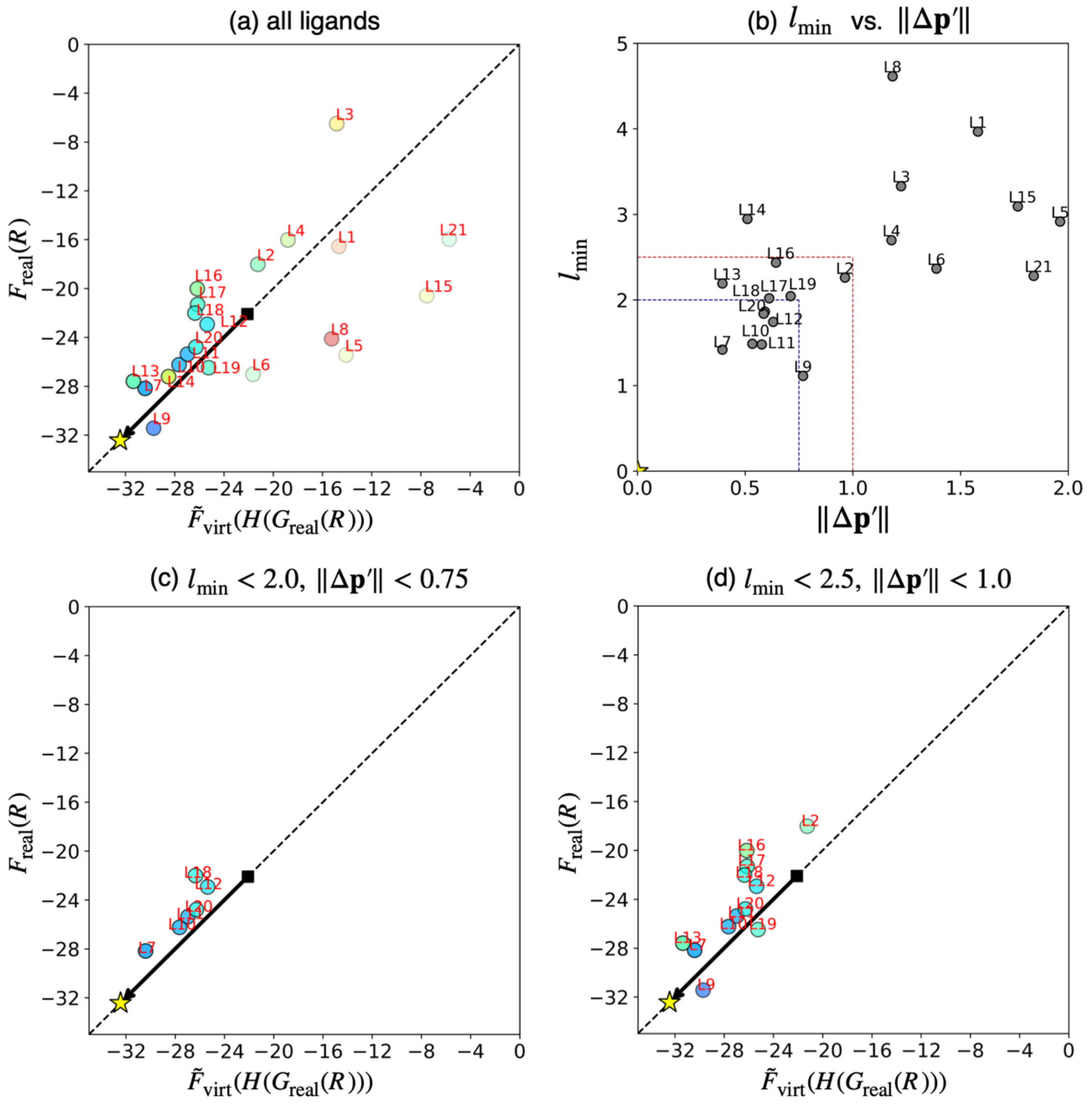

Figure S2. Internal validation of the prediction algorithm. The objective value ΔE was set as the difference in ligand dissociation energies between $R_3PPd(PH_3)_3$ and $R_3PCr(CO)_5$. In (a), (c) and (d), $F_{real}(R)$ and $\tilde{F}_{virt}(H(G_{real}(R)))$ correspond to the calculated and predicted ΔE values in kcal/mol, respectively.

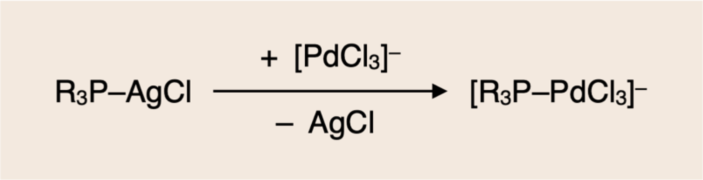

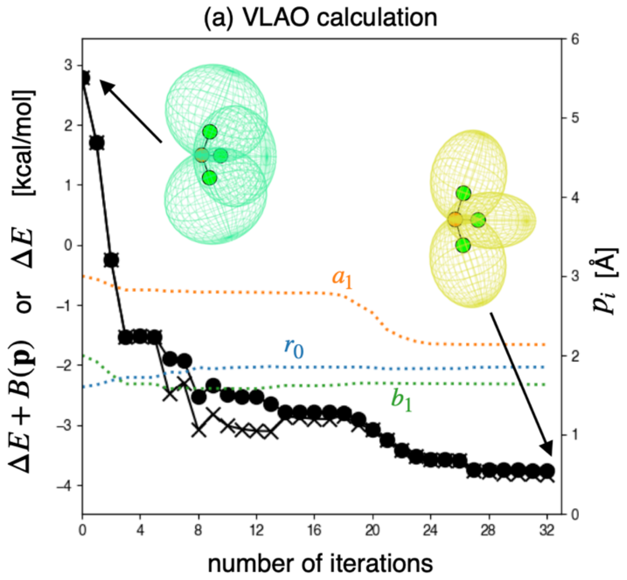

(b) Gradient and Hessian

$$\frac{\mathrm{d}\Delta E}{\mathrm{d}\mathbf{p}} = \left(\frac{\partial \Delta E}{\partial r_0}, \frac{\partial \Delta E}{\partial a_1}, \frac{\partial \Delta E}{\partial b_1}\right) = (-11.5,\ 1{,}3,\ 2.2)$$

kcal/(mol•Å)

$$\frac{\mathrm{d}^2\Delta E}{\mathrm{d}\mathbf{p}^2} = \begin{pmatrix} -42.7 & 0.1 & -0.6 \\ 0.1 & 1.4 & 5.2 \\ -0.6 & 5.2 & 2.8 \end{pmatrix}$$

kcal/(mol•Å^2)

Figure S3. The detailed results of VLAO calculation to minimize $\Delta E + B(\mathbf{p})$, where ΔE represents the difference in ligand dissociation energies between R_3PAgCl and $[R_3PPdCl_3]^-$.

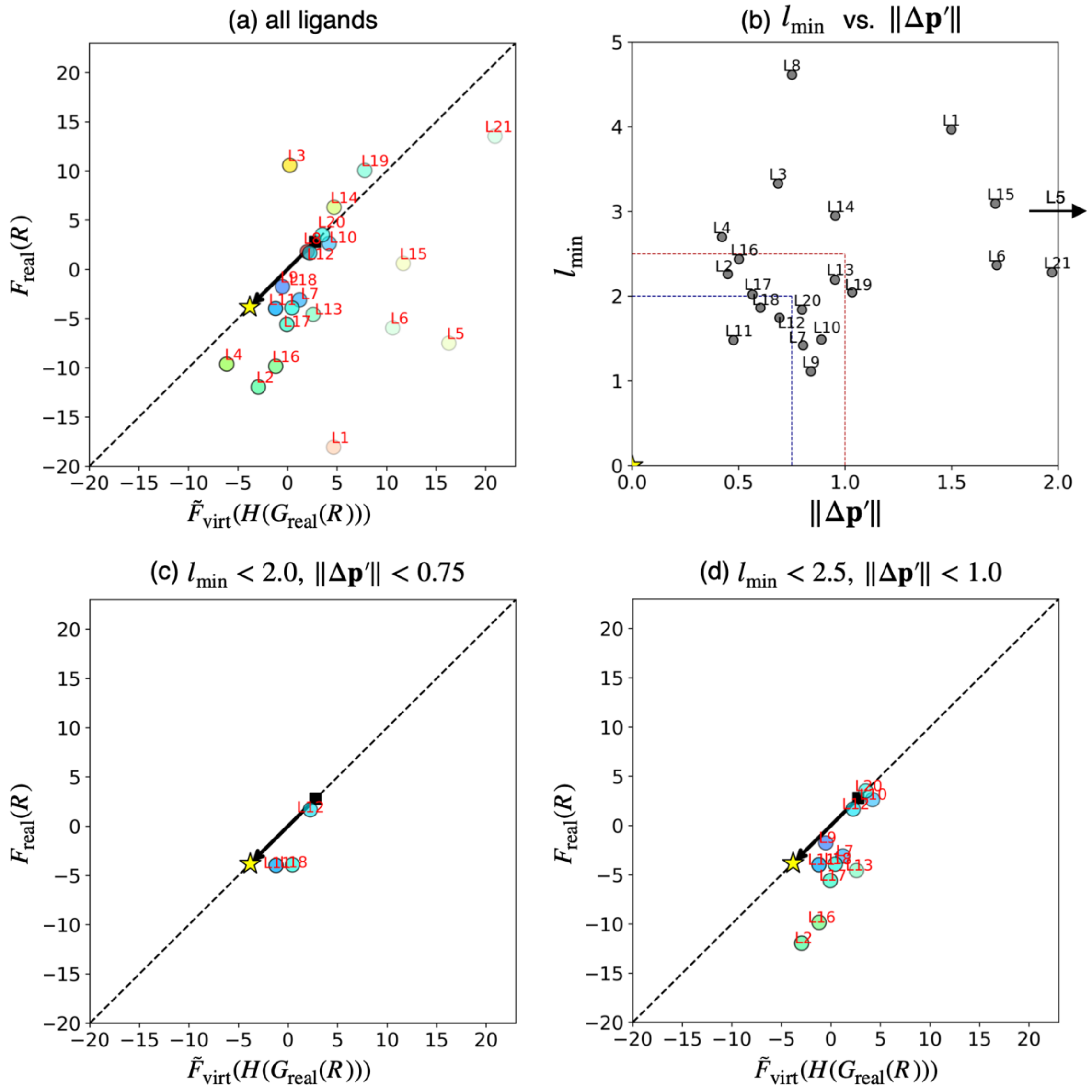

Figure S4. Internal validation of the prediction algorithm. The objective value ΔE was set as the difference in ligand dissociation energies between R_3PAgCl and $[R_3PPdCl_3]^-$. In (a), (c) and (d), $F_{real}(R)$ and $\tilde{F}_{virt}(H(G_{real}(R)))$ correspond to the calculated and predicted ΔE values in kcal/mol, respectively.

4. Detailed results of external validations

The detailed results of the external validations were shown in Figure S5–S10. In the VLAO calculation, the parameter ranges of the barrier function $B(\mathbf{p})$ was set to 1.3–1.9 Å for r_0, 2.0–3.5 Å for a_1, and 1.5–3.0 Å for b_1.

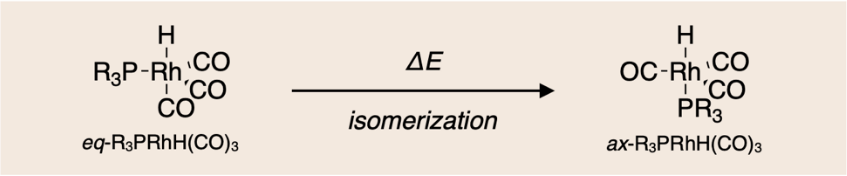

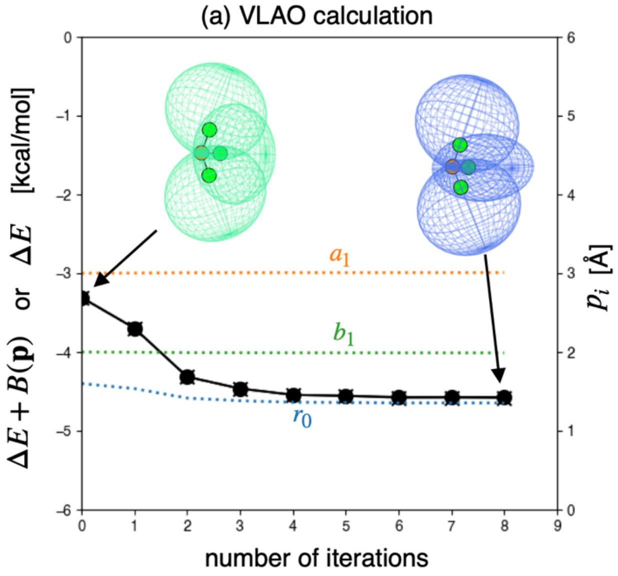

(b) Gradient and Hessian

$$\frac{\mathrm{d}\Delta E}{\mathrm{d}\mathbf{p}} = \left(\frac{\partial \Delta E}{\partial r_0}, \frac{\partial \Delta E}{\partial a_1}, \frac{\partial \Delta E}{\partial b_1}\right) = (4.1, \ -0.2, 0.3)$$

kcal/(mol•Å)

$$\frac{\mathrm{d}^2\Delta E}{\mathrm{d}\mathbf{p}^2} = \begin{pmatrix} 10.4 & -0.1 & -0.2 \\ -0.1 & -0.4 & 0.4 \\ -0.2 & 0.4 & -0.03 \end{pmatrix}$$

kcal/(mol•Å²)

Figure S5. The detailed results of VLAO calculation to minimize $\Delta E + B(\mathbf{p})$, where ΔE represents the difference in ligand dissociation energies between *eq*-$R_3PRhH(CO)_3$ and *ax*-$R_3PRhH(CO)_3$.

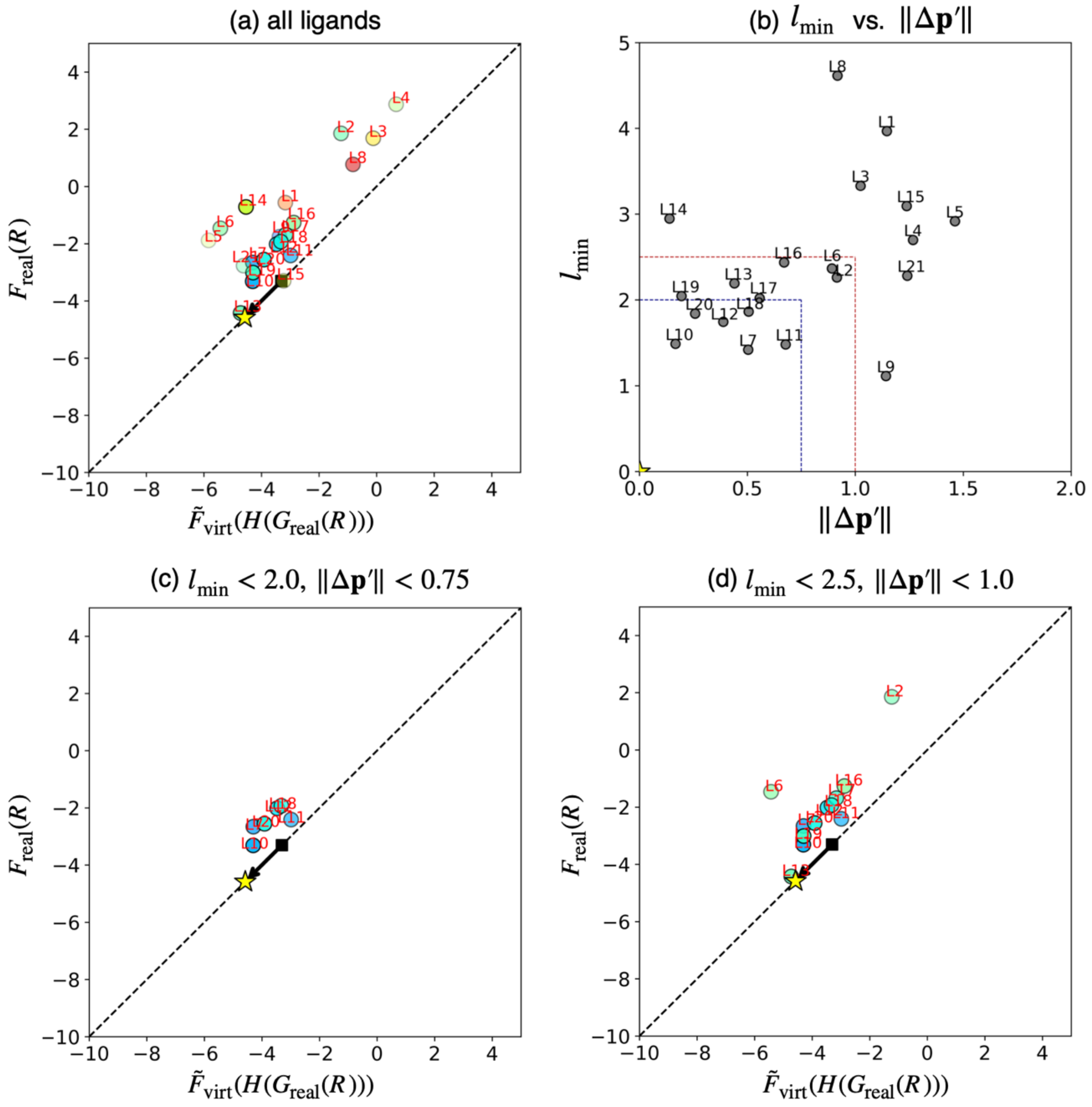

Figure S6. External validation of the prediction algorithm. The objective value ΔE was set as the difference in ligand dissociation energies between *eq*-$R_3PRhH(CO)_3$ and *ax*-$R_3PRhH(CO)_3$. In (a), (c) and (d), $F_{\mathrm{real}}(R)$ and $\tilde{F}_{\mathrm{virt}}(H(G_{\mathrm{real}}(R)))$ correspond to the calculated and predicted ΔE values in kcal/mol, respectively.

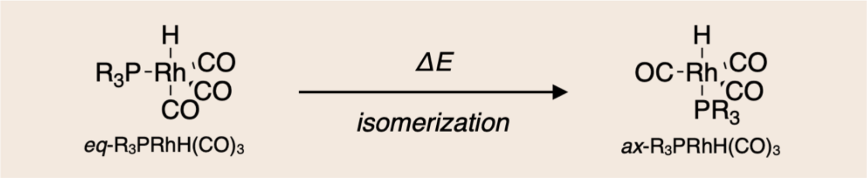

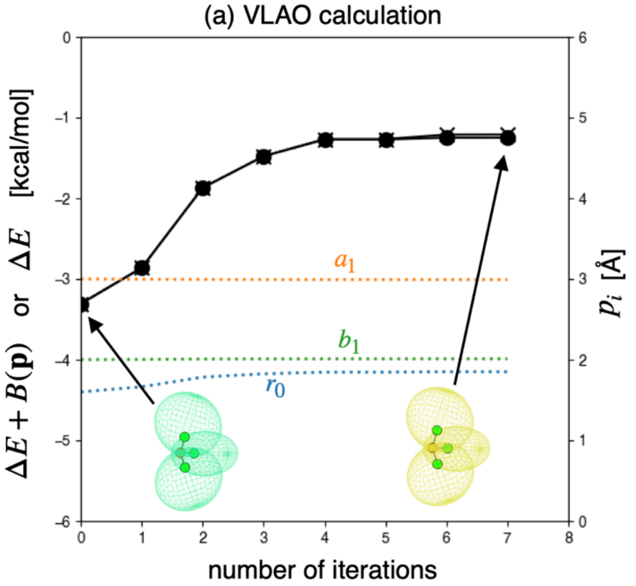

(b) Gradient and Hessian

$$\frac{\mathrm{d}\Delta E}{\mathrm{d}\mathbf{p}} = \left(\frac{\partial \Delta E}{\partial r_0}, \frac{\partial \Delta E}{\partial a_1}, \frac{\partial \Delta E}{\partial b_1}\right) = (10.6, \ -0.2, 0.3)$$

kcal/(mol•Å)

$$\frac{\mathrm{d}^2\Delta E}{\mathrm{d}\mathbf{p}^2} = \begin{pmatrix} 23.4 & -0.2 & -0.1 \\ -0.2 & -0.4 & 0.4 \\ -0.1 & 0.4 & -0.1 \end{pmatrix}$$

kcal/(mol•Å^2)

Figure S7. The detailed results of VLAO calculation to maximize $\Delta E - B(\mathbf{p})$, where ΔE represents the difference in ligand dissociation energies between *eq*-$R_3PRhH(CO)_3$ and *ax*-$R_3PRhH(CO)_3$.

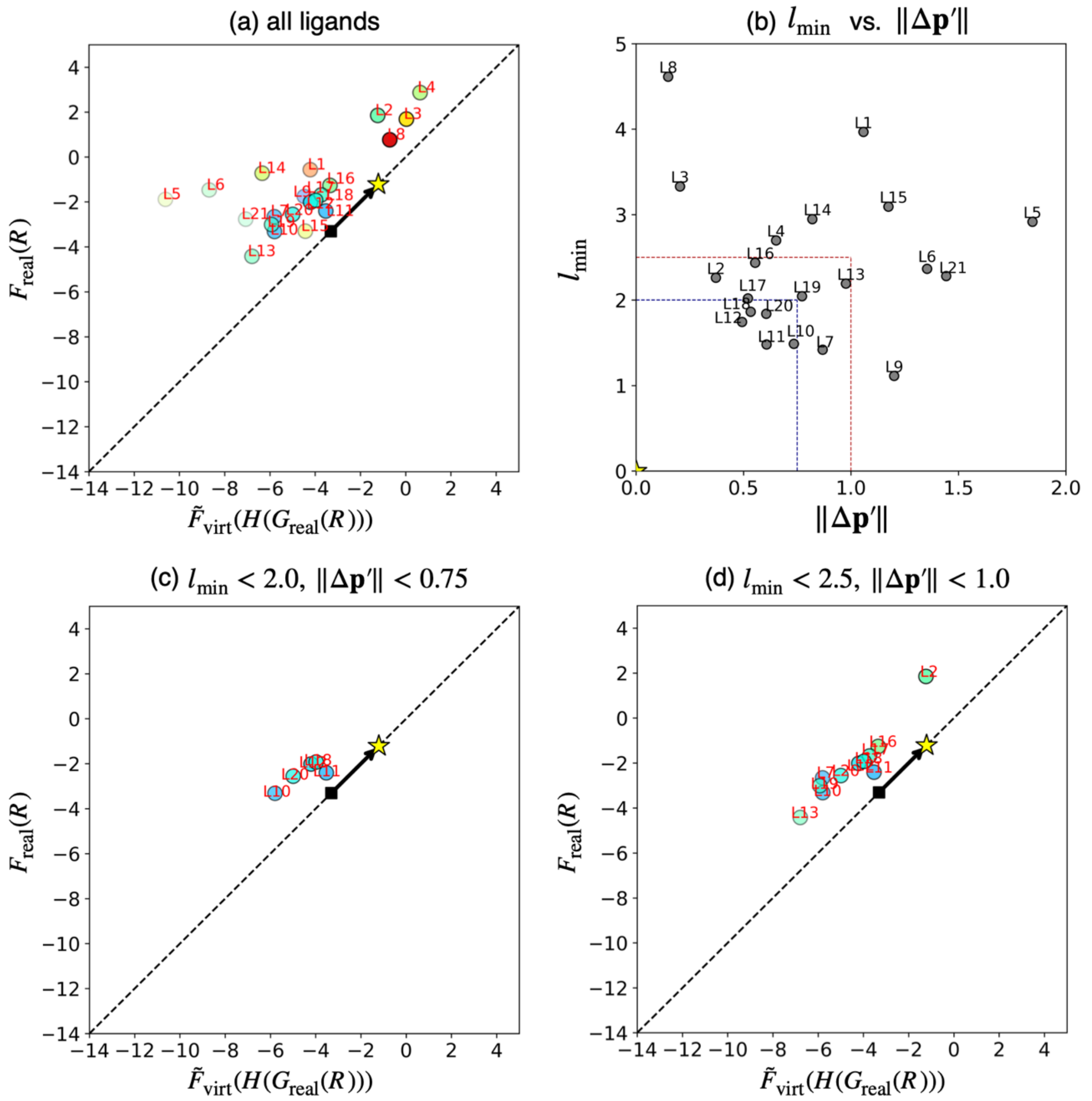

Figure S8. External validation of the prediction algorithm. The objective value ΔE was set as the difference in ligand dissociation energies between *eq*-$R_3PRhH(CO)_3$ and *ax*-$R_3PRhH(CO)_3$. In (a), (c) and (d), $F_{real}(R)$ and $\tilde{F}_{virt}(H(G_{real}(R)))$ correspond to the calculated and predicted ΔE values in kcal/mol, respectively.

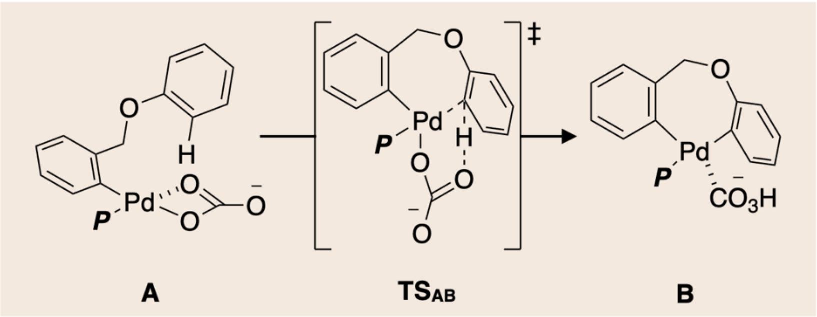

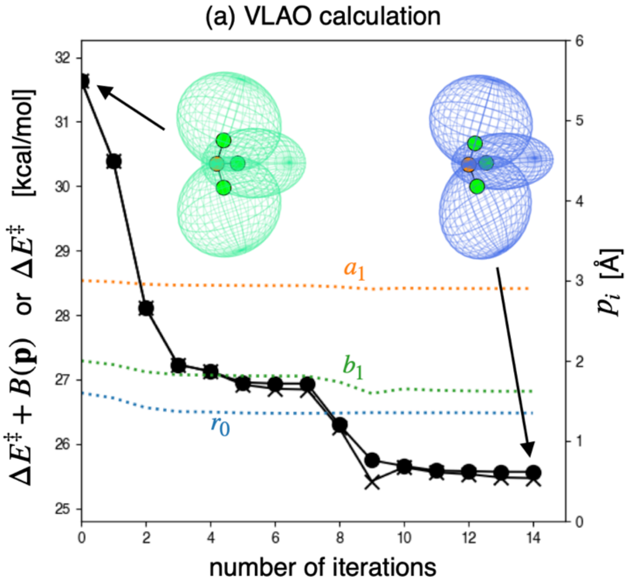

(b) Gradient and Hessian

$$\frac{\mathrm{d}\Delta E^{\ddagger}}{\mathrm{d}\mathbf{p}} = \left(\frac{\partial \Delta E^{\ddagger}}{\partial r_0}, \frac{\partial \Delta E^{\ddagger}}{\partial a_1}, \frac{\partial \Delta E^{\ddagger}}{\partial b_1}\right) = (11.0, \ -0.7, \ 4.2)$$

kcal/(mol•Å)

$$\frac{\mathrm{d}^2\Delta E^{\ddagger}}{\mathrm{d}\mathbf{p}^2} = \begin{pmatrix} 11.7 & 2.6 & 5.0 \\ 2.6 & 1.8 & 8.7 \\ 5.0 & 8.7 & 30.3 \end{pmatrix}$$

kcal/(mol•Å^2)

Figure S9. The detailed results of VLAO calculation to minimize $\Delta E^{\ddagger} + B(\mathbf{p})$, where $\Delta E^{\ddagger}$ represents the activation energy of the reaction.

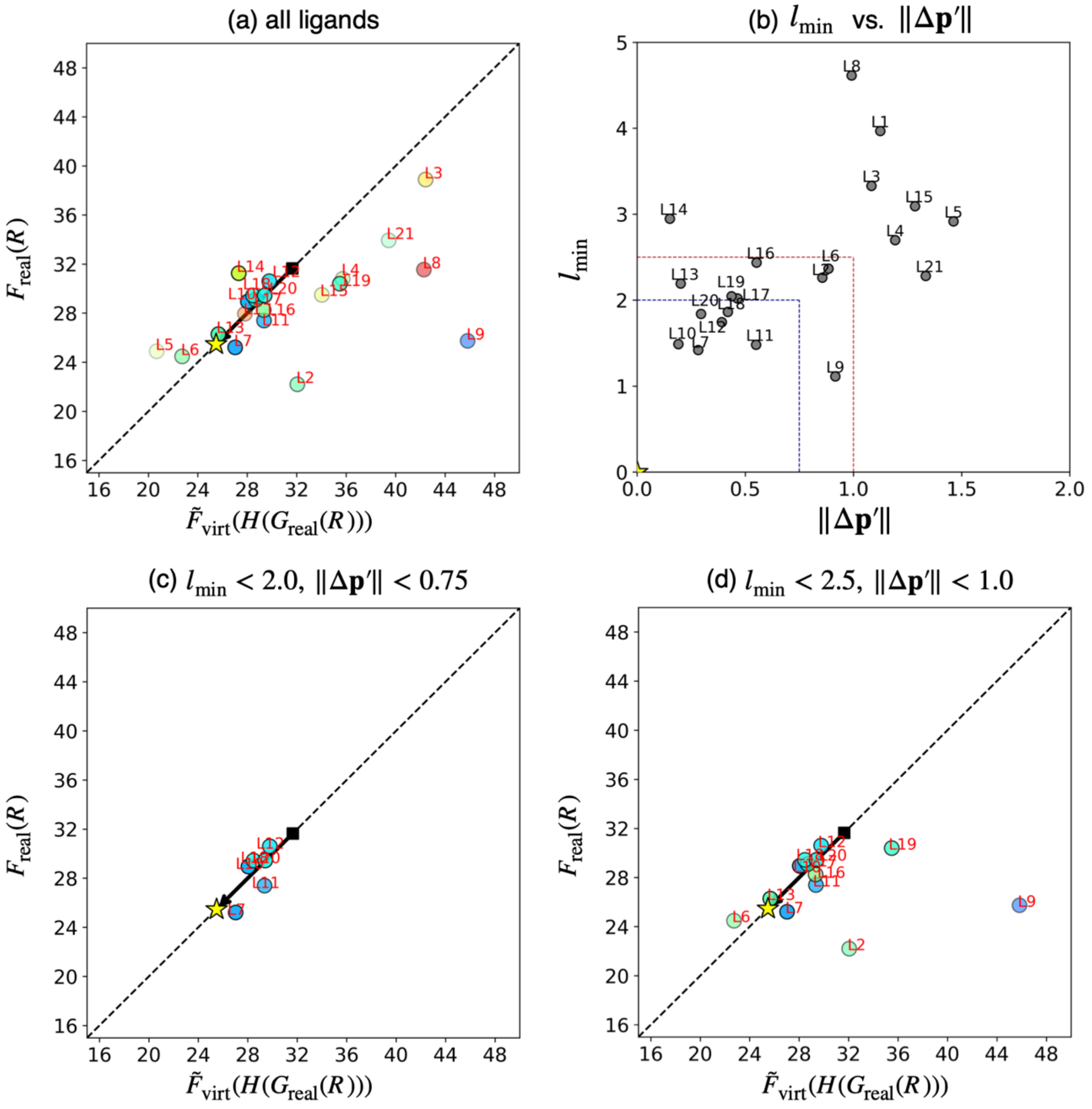

Figure S10. External validation of the prediction algorithm. The objective value $\Delta E^{\ddagger}$ was set as the activation energy of the reaction. In (a), (c) and (d), $F_{real}(R)$ and $\tilde{F}_{virt}(H(G_{real}(R)))$ correspond to the calculated and predicted $\Delta E^{\ddagger}$ values in kcal/mol, respectively.

5. Results with expanded parameter ranges

The results of the external validations with expanded parameter ranges of $B(\mathbf{p})$ were shown in Figures S11–S15. The parameter ranges were set to 1.0–2.2 Å for r_0, 1.5–5.5 Å for a_1, and 0.5–2.5 Å for b_1.

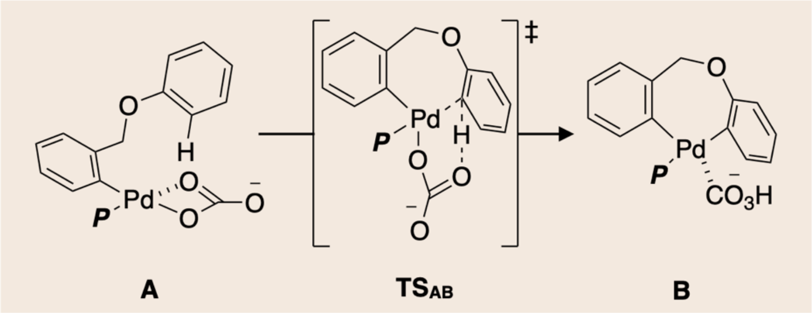

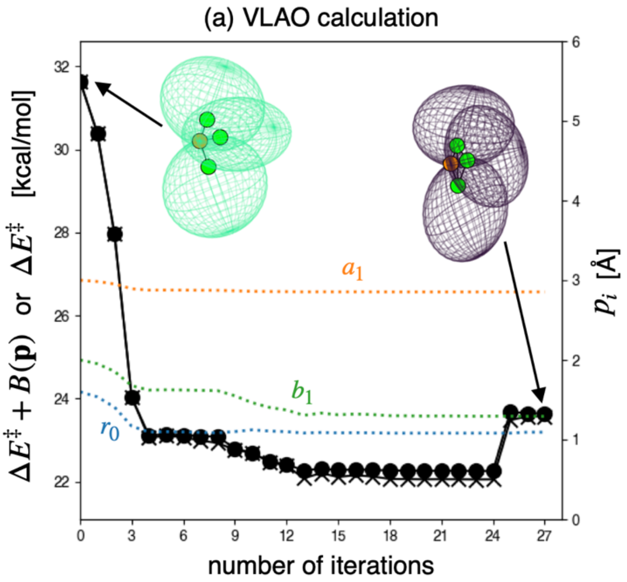

(b) Gradient and Hessian

$$\frac{\mathrm{d}\Delta E^{\ddagger}}{\mathrm{d}\mathbf{p}} = \left(\frac{\partial \Delta E^{\ddagger}}{\partial r_0}, \frac{\partial \Delta E^{\ddagger}}{\partial a_1}, \frac{\partial \Delta E^{\ddagger}}{\partial b_1}\right) = (8{,}1,\ -0.3,\ 0.7)$$

kcal/(mol•Å)

$$\frac{\mathrm{d}^2\Delta E^{\ddagger}}{\mathrm{d}\mathbf{p}^2} = \begin{pmatrix} 83.2 & 8.8 & 24.7 \\ 8.8 & 1.1 & 1.9 \\ 24.7 & 1.9 & 3.1 \end{pmatrix}$$

kcal/(mol•Å^2)

Figure S11. The detailed results of VLAO calculation to minimize $\Delta E^{\ddagger} + B(\mathbf{p})$, where $\Delta E^{\ddagger}$ represents the activation energy of the reaction.

Note that the objective function ($\Delta E^{\ddagger} + B(\mathbf{p})$) increased by approximately 1.5 kcal/mol at the 25th iteration and then converged without returning to its previous value. This was due to the conformational change of **A** at the 25th iteration, where the geometry optimization converged to a more stable conformation through the rotation of the Pd–P bond.

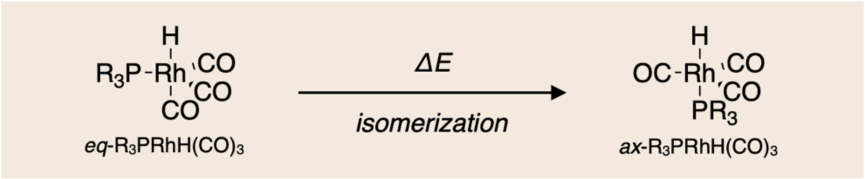

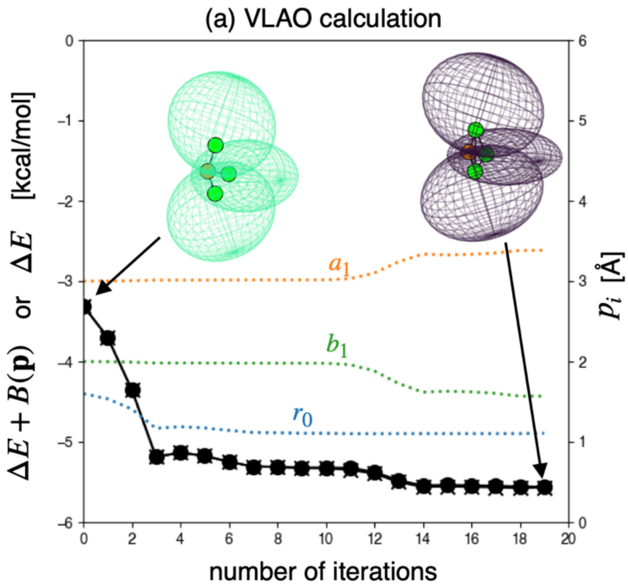

(b) Gradient and Hessian

$$\frac{\mathrm{d}\Delta E}{\mathrm{d}\mathbf{p}} = \left(\frac{\partial \Delta E}{\partial r_0}, \frac{\partial \Delta E}{\partial a_1}, \frac{\partial \Delta E}{\partial b_1}\right) = (2.3, \ -0.5, \ -0.3)$$

kcal/(mol•Å)

$$\frac{\mathrm{d}^2\Delta E}{\mathrm{d}\mathbf{p}^2} = \begin{pmatrix} 2.5 & -0.03 & 0.8 \\ -0.03 & -0.1 & -0.5 \\ 0.8 & -0.5 & 3.2 \end{pmatrix}$$

kcal/(mol•Å^2)

Figure S12. The detailed results of VLAO calculation to minimize $\Delta E + B(\mathbf{p})$, where ΔE represents the difference in ligand dissociation energies between *eq*-$R_3PRhH(CO)_3$ and *ax*-$R_3PRhH(CO)_3$.

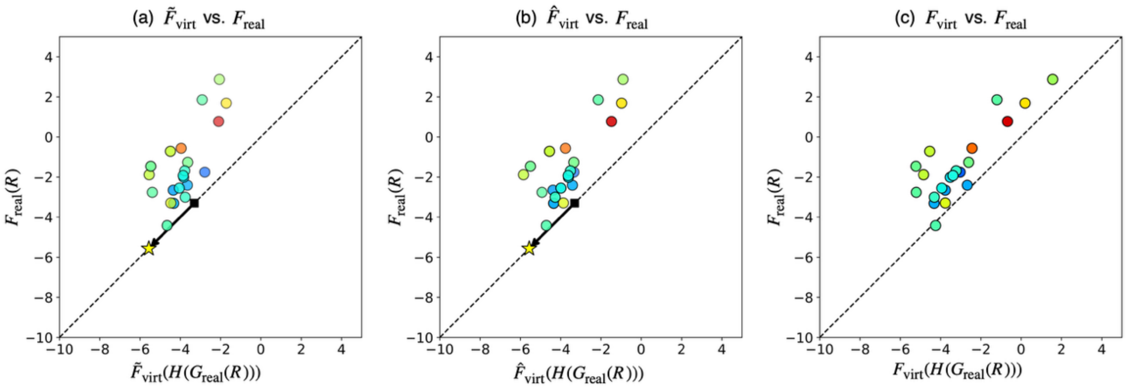

Figure S13. Comparison of model functions to approximate F_{virt}. The objective value ΔE was set as the difference in ligand dissociation energies between *eq*-$R_3PRhH(CO)_3$ and *ax*-$R_3PRhH(CO)_3$. $F_{\mathrm{real}}(R)$ corresponds to the calculated ΔE values in kcal/mol. $\tilde{F}_{\mathrm{virt}}\big(H(G_{\mathrm{real}}(R))\big)$, $\hat{F}_{\mathrm{virt}}\big(H(G_{\mathrm{real}}(R))\big)$, $F_{virt}\big(H(G_{\mathrm{real}}(R))\big)$ correspond to the predicted ΔE values in kcal/mol.

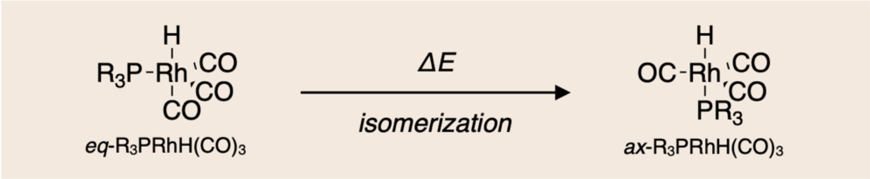

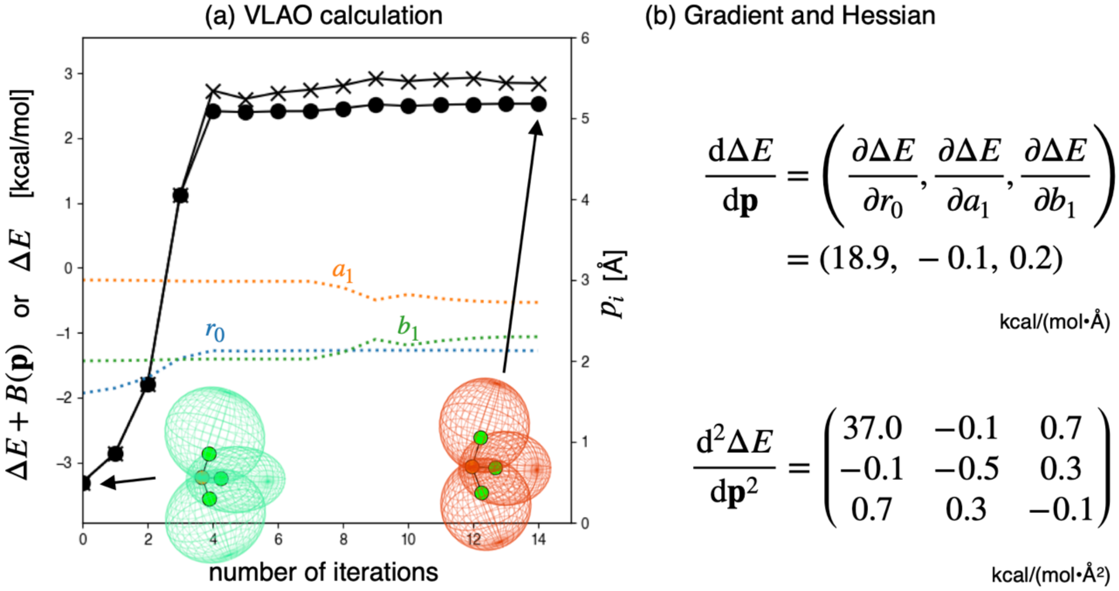

Figure S14. The detailed results of VLAO calculation to maximize $\Delta E - B(\mathbf{p})$, where ΔE represents the difference in ligand dissociation energies between *eq*-$R_3PRhH(CO)_3$ and *ax*-$R_3PRhH(CO)_3$.

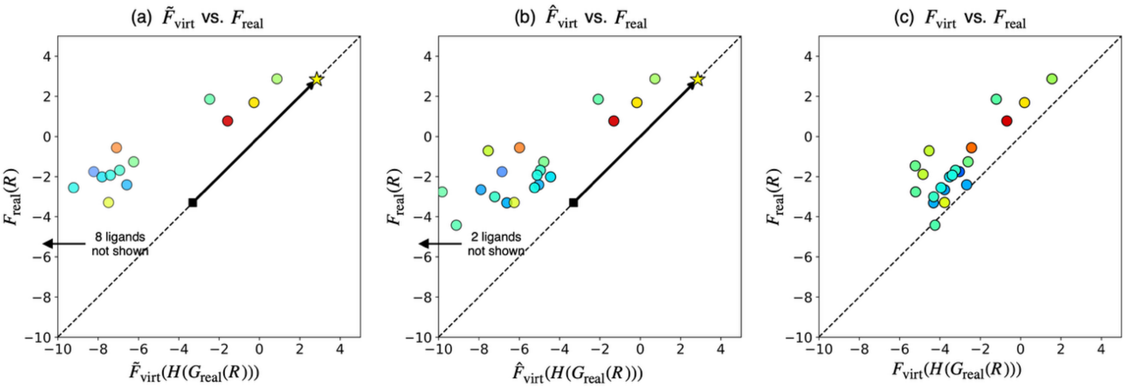

Figure S15. Comparison of model functions to approximate F_{virt}. The objective value ΔE was set as the difference in ligand dissociation energies between *eq*-$R_3PRhH(CO)_3$ and *ax*-$R_3PRhH(CO)_3$. $F_{\mathrm{real}}(R)$ corresponds to the calculated ΔE values in kcal/mol. $\tilde{F}_{\mathrm{virt}}(H(G_{\mathrm{real}}(R)))$, $\hat{F}_{\mathrm{virt}}(H(G_{\mathrm{real}}(R)))$, $F_{virt}(H(G_{\mathrm{real}}(R)))$ correspond to the predicted ΔE values in kcal/mol.

6. Additional internal validations

To further assess the generality of the present method, we performed three additional internal validation following the same protocol as described in the main text (Figures 7–9), with the results summarized in Figure S16. These validation sets were specifically designed to emphasize diversity in the transition metal center, in order to test the method across a broad catalyst space. In all cases, we observed good agreement between the predicted and computed values, particularly for ligands with high reliability metrics. Taken together with the main text results, these findings support the applicability of the proposed approach to a wide range of metal centers, including Cr, Cu, Ag, Au, Ni, Pd, Pt, Rh, and Ir.

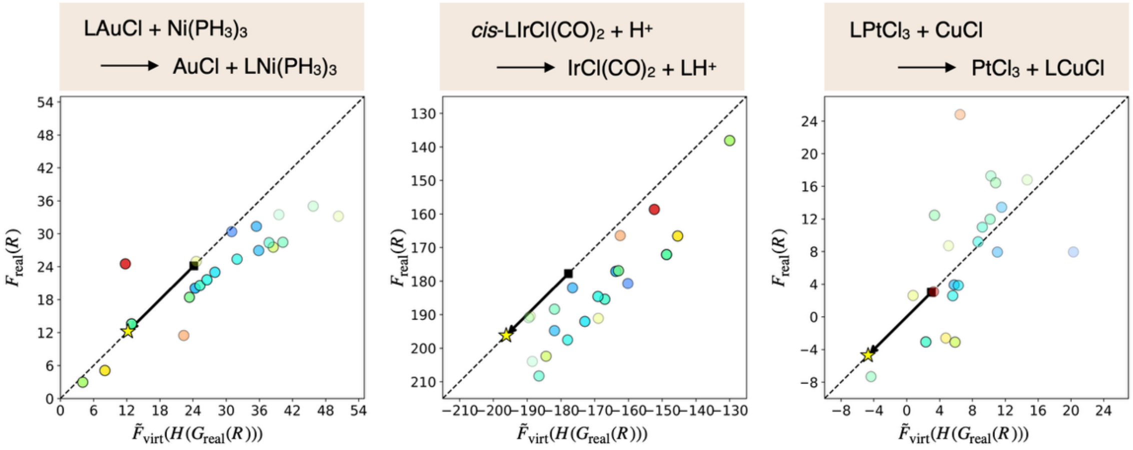

Figure S16. Additional internal validation of the prediction algorithm. The objective value was set as the difference in ligand dissociation energies between (a) LAuCl and $LNi(PH_3)_3$, (b) cis-$LirCl(CO)_2$ and H^+ and (c) $LPtCl_3$ and LCuCl. $F_{\mathrm{real}}(R)$ and $\tilde{F}_{\mathrm{virt}}(H(G_{\mathrm{real}}(R)))$ correspond to the calculated and predicted $\Delta E^{\ddagger}$ values in kcal/mol, respectively.

7. Attempts Using Conventional Prediction Methods

To benchmark the present method against conventional data-driven approaches, we constructed regression models using computational data for the C–H activation on palladium(II) complexes (Scheme 1 in the main text). For each of the 21 ligands, 194 descriptors were extracted from Kraken.[S7] Regression models were then built using Lasso and Ridge regression (LassoCV, RidgeCV from scikit-learn[S8]), as well as Random Forest regression (RandomForestRegressor).

Figure S17 presents the model performance both for in-sample predictions (*i.e.*, predictions on the training data) and for leave-one-out (LOO) cross-validation, where each activation barrier was predicted using a model trained on the remaining 20 data points. While all models yielded high R^2 values and low mean squared errors (MSEs) for the in-sample predictions, they failed to produce statistically meaningful results under LOO validation. This outcome suggests that the current dataset size (21 samples) is insufficient for building reliable predictive models using standard regression techniques. We also explored preselection of descriptors based on correlation and variance filtering, but the results remained essentially unchanged.

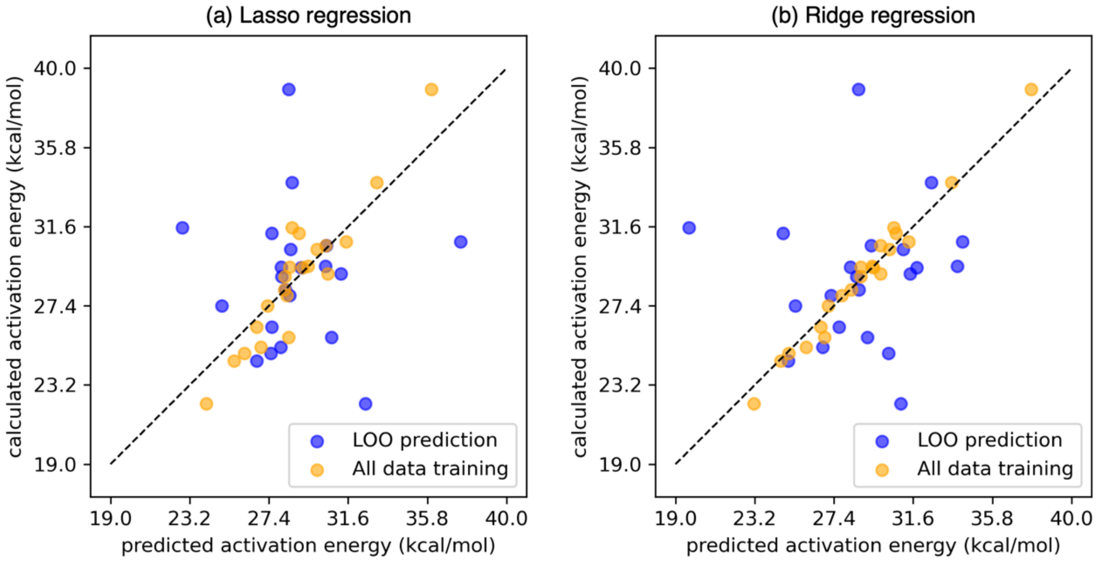

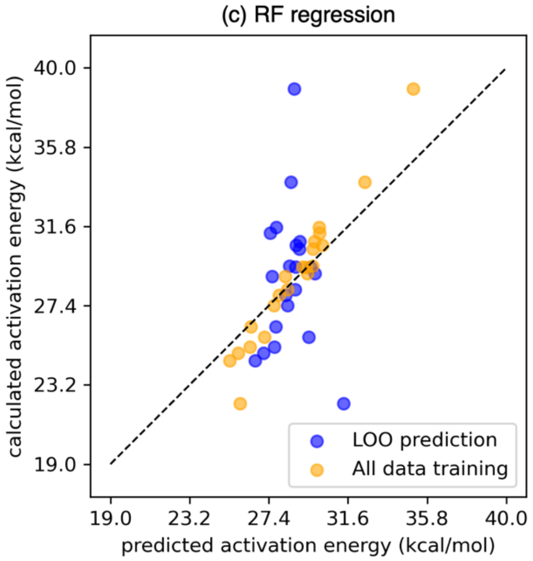

model	training	R^2	MSE (kcal^2/mol^2)
Lasso	all data	0.833	2.09 ± 2.79
Lasso	LOO	-0.696	21.1 ± 33.5
Ridge	all data	0.972	0.351 ± 0.404
Ridge	LOO	-0.764	22.0 ± 37.5
RF	all data	0.843	1.96 ± 4.02
RF	LOO	-0.106	13.8 ± 26.9

Figure S17. Prediction models using standard regression techniques. Orange and blue circles denote in-sample and LOO predictions, respectively.

8. Supplemental references

S1. Gaussian 16, Revision C.01, Frisch, M. J.; Trucks, G. W.; Schlegel, H. B.; Scuseria, G. E.; Robb, M. A.; Cheeseman, J. R.; Scalmani, G.; Barone, V.; Petersson, G. A.; Nakatsuji, H.; Li, X.; Caricato, M.; Marenich, A. V.; Bloino, J.; Janesko, B. G.; Gomperts, R.; Mennucci, B.; Hratchian, H. P.; Ortiz, J. V.; Izmaylov, A. F.; Sonnenberg, J. L.; Williams-Young, D.; Ding, F.; Lipparini, F.; Egidi, F.; Goings, J.; Peng, B.; Petrone, A.; Henderson, T.; Ranasinghe, D.; Zakrzewski, V. G.; Gao, J.; Rega, N.; Zheng, G.; Liang, W.; Hada, M.; Ehara, M.; Toyota, K.; Fukuda, R.; Hasegawa, J.; Ishida, M.; Nakajima, T.; Honda, Y.; Kitao, O.; Nakai, H.; Vreven, T.; Throssell, K.; Montgomery, J. A., Jr.; Peralta, J. E.; Ogliaro, F.; Bearpark, M. J.; Heyd, J. J.; Brothers, E. N.; Kudin, K. N.; Staroverov, V. N.; Keith, T. A.; Kobayashi, R.; Normand, J.; Raghavachari, K.; Rendell, A. P.; Burant, J. C.; Iyengar, S. S.; Tomasi, J.; Cossi, M.; Millam, J. M.; Klene, M.; Adamo, C.; Cammi, R.; Ochterski, J. W.; Martin, R. L.; Morokuma, K.; Farkas, O.; Foresman, J. B.; Fox, D. J. Gaussian, Inc., Wallingford CT, 2016.

S2. (a) Grimme, S.; Bannwarth, C.; Shushkov, P. A Robust and Accurate Tight-Binding Quantum Chemical Method for Structures, Vibrational Frequencies, and Noncovalent Interactions of Large Molecular Systems Parametrized for All spd-Block Elements (Z = 1–86). *J. Chem. Theory Comput.* **2017**, *13*, 1989–2009. (b) Grimme, D.; Bannwarth, C. Ultra-fast Computation of Electronic Spectra for Large Systems by Tight-Binding Based Simplified Tamm-Dancoff Approximation (sTDA-xTB). *J. Chem. Phys.* **2016**, *145*, 054103.

S3. Neese, F.; Wennmohs, F.; Becker, U.; Riplinger, C. The ORCA Quantum Chemistry Program Package. *J. Chem. Phys.* **2020**, *152*, 224108.

S4. Maeda, S.; Harabuchi, Y.; Takagi, M.; Saita, K.; Suzuki, K.; Ichino, T.; Sumiya, Y.; Sugiyama, K.; Ono, Y. Implementation and Performance of the Artificial Force Induced Reaction Method in the GRRM17 Program. *J. Comput. Chem.* **2018**, *39*, 233–250.

S5. Matsuoka, W.; Oki, T.; Yamada, R.; Yokoyama, T.; Suda, S.; Saunders, C. M.; Skjelstad, B. B.; Harabuchi, Y.; Fey, N.; Iwata, S.; Maeda, S. Virtual Ligand-Assisted Optimization: A Rational Strategy for Ligand Engineering. *ACS Catal.* **2024**, *14*, 16297–16312.

S6. Paszke, A.; Gross, S.; Massa, F.; Lerer, A.; Bradbury, J.; Chanan, G.; Killeen, T.; Lin, Z.; Gimelshein, N.; Antiga, L.; Desmaison, A.; Kopf, A.; Yang, E.; DeVito, Z.; Raison, M.; Tejani, A.; Chilamkurthy, S.; Steiner, B.; Fang, L.; Bai, J.; Chintala, S. PyTorch: An Imperative Style, High-Performance Deep Learning Library. *Advances in Neural Information Processing Systems* **2019**, *32*, 8024–8035.

S7. Gensch, T.; dos Passos Gomes, G.; Friederich, P.; Peters, E.; Gaudin, T.; Pollice, R.; Jorner, K.; Nigam, A.; Lindner-D'Addario, M.; Sigman, M. S.; Aspuru-Guzik, A. A Comprehensive Discovery Platform for Organophosphorus Ligands for Catalysis. *J. Am. Chem. Soc.* **2022**, *144*, 1205–1217.

S8. Pedregosa, F.; Varoquaux, G.; Gramfort, A.; Michel, V.; Thirion, B.; Grisel, O.; Blondel, M.; Prettenhofer, P.; Weiss, R.; Dubourg, V.; Vanderplas, J.; Passos, A.; Cournapeau, D.; Brucher, M.; Perrot, M.; Duchesnay, É. Scikit-learn: Machine Learning in Python. *J. Mach. Learn. Res.* **2011**, *12*, 2825–2830.
